# Engineering energetically efficient transport of dicarboxylic acids in yeast *Saccharomyces cerevisiae*

**DOI:** 10.1073/pnas.1900287116

**Published:** 2019-08-29

**Authors:** Behrooz Darbani, Vratislav Stovicek, Steven Axel van der Hoek, Irina Borodina

**Affiliations:** ^a^The Novo Nordisk Foundation Center for Biosustainability, Technical University of Denmark, 2800 Kgs. Lyngby, Denmark

**Keywords:** cell factories, dicarboxylic acids, efflux transporters, *Schizosaccharomyces pombe* MAE1, SLAC1

## Abstract

Biobased C4-dicarboxylic acids are attractive sustainable precursors for polymers and other materials. Commercial scale production of these acids at high titers requires efficient secretion by cell factories. In this study, we characterized 7 dicarboxylic acid transporters in *Xenopus* oocytes and in *Saccharomyces cerevisiae* engineered for dicarboxylic acid production. Among the tested transporters, the Mae1(p) from *Schizosaccharomyces pombe* had the highest activity toward succinic, malic, and fumaric acids and resulted in 3-, 8-, and 5-fold titer increases, respectively, in *S. cerevisiae*, while not affecting growth, which was in contrast to the tested transporters from the tellurite-resistance/dicarboxylate transporter (TDT) family or the Na^+^ coupled divalent anion–sodium symporter family. Similar to *Sp*Mae1(p), its homolog in *Aspergillus carbonarius, Ac*Dct(p), increased the malate titer 12-fold without affecting the growth. Phylogenetic and protein motif analyses mapped *Sp*Mae1(p) and *Ac*Dct(p) into the voltage-dependent slow-anion channel transporter (SLAC1) clade of transporters, which also include plant Slac1(p) transporters involved in stomata closure. The conserved phenylalanine residue F329 closing the transport pore of *Sp*Mae1(p) is essential for the transporter activity. The voltage-dependent SLAC1 transporters do not use proton or Na^+^ motive force and are, thus, less energetically expensive than the majority of other dicarboxylic acid transporters. Such transporters present a tremendous advantage for organic acid production via fermentation allowing a higher overall product yield.

## Significance

The export of organic acids is typically proton or sodium coupled and requires energetic expenditure. Consequently, the cell factories producing organic acids must use part of the carbon feedstock on generating the energy for export, which decreases the overall process yield. Here, we show that organic acids can be exported from yeast cells by voltage-gated anion channels without the use of proton, sodium, or ATP motive force, resulting in more efficient fermentation processes.

Microbial cell factories are designed to convert cheap substrates into added-value products, such as fuels, commodity, specialty chemicals, and drugs. It is usually advantageous if the cells efficiently secrete the products into the medium. This minimizes feedback inhibition and toxicity, prevents product degradation, and facilitates downstream processing (1, 2).

Efflux engineering has improved the production of antibiotics griseoviridin and viridogrisein in *Streptomyces griseoviridis* (3), itaconic acid production in *Ustilago vetiveriae* and *Aspergillus terreus* (4, 5), amorphadiene (6), butanol (7), isopentenol (8), and flavins (9) production in *Escherichia coli*. In yeast *S. cerevisiae*, there are examples, where improved secretion was engineered for short branched-chain fatty acids (10), ethanol (11, 12), and alkanes (13). Due to the high industrial relevance, the transport of organic acids, particularly dicarboxylic acids, is one of the most studied and yet poorly understood phenomena.

C4-dicarboxylic acids, such as succinic, malic, and fumaric acid, are widely used in food, pharmaceutical, and polymers industries (14). These acids are primarily derived from petroleum and gas, however, recently novel processes for production of succinic and malic acids by fermentation have been commercialized where several processes apply yeast as the cell factory (e.g., Reverdia, BioAmber). To improve the secretion of dicarboxylic acids by the producing cells, various transporters have been expressed in bacteria (15–18). In yeasts, the transporter Mae1(p) from malate-utilizing yeast *S. pombe* was applied to increase the secretion of succinic and malic acids in multiple studies (19, 20). In this study, we uncover the mechanism of action of this transporter and show that it likely belongs to the SLAC1 family, similar to the Slac1(p) transporters in plants that participate in stomatal closure. We hypothesize that the superior performance of *Sp*Mae1(p) is likely due to the low energy requirement for organic acids export.

## Results

### Selection of Candidate Dicarboxylic Acid Transporters.

We examined several candidates of carboxylic acid transporter genes from different organisms (Table 1). We included the Mae1(p) from *S. pombe*, previously shown to be effective for malate and succinate production in *S. cerevisiae* (21–23) and its homolog in the natural malic acid producer *Aspergillus oryzae Ao*Mae1. Interestingly, the *AoMAE1* gene is colocalized with the succinyl-CoA ligase gene, involved in the tricarboxylic acid cycle (*SI Appendix*, Fig. S1). We also selected *S. cerevisiae* mitochondrial citrate transporter Ctp1(p). From the bacterial transporters, we chose two dicarboxylate transporters *Ec*Dcuc(p) and *Ec*Dcub(p) from *E. coli* (17) and a homolog of *Ec*Dcub(p), a putative transporter called *As*Dct(p) from the succinate producer *Actinobacillus succinogenes*. The genes encoding *Ec*Dcuc(p) and *Ec*Dcub(p) in *E. coli* are colocalized with the fumarase B gene and with genes involved in citrate utilization. Finally, we found a putative transporter SLC13(p) that was colocalized with succinyl-CoA synthetase and oxoglutarate dehydrogenase genes in *A. succinogenes*. The selected 7 transporter candidates were phylogenetically classified into 5 transporter families (Table 1). For Mae1(p) transporters, homologs could be found only in fungi and plant kingdoms, while for the DCU and DCUC families, the homologs were found in animals and fungi but not in plants. Several of the selected transporter genes were adjacent to related pathway genes, and these phenomena can be exploited for transporter function prediction as illustrated previously for secondary metabolites in bacteria and plants (24, 25).

**Table 1.** Dicarboxylic acid membrane transporters

Transporter*	Transporter Family/Class	Homologs^†^
*S. cerevisiae* Mae1	TDT/2.A.16	Plant
*A. oryzae* Mae1	TDT/2.A.16	Plant
*S. cerevisiae* Ctp1	Mitochondrial Carrier/2.A.29	Animal, plant
*A. succinogenes* Dct	DiCarboxylate Uptake/2.A.13	Animal
*A. succinogenes* Slc13	Divalent anion:Na^+^ symporter/2.A.47	Animal, plant, Fungi
*E. coli* Dcub	DiCarboxylate uptake/2.A.13	Animal
*E. coli* Dcuc	DiCarboxylate uptakeC/2.A.61	Animal, ungi

* Sequence accession numbers are provided in *SI Appendix*, Table S6.

^†^ Homologs from other kingdoms, if any, retrieved as the immediate hit from National Center for Biotechnology Information protein blast.

### Expression of the Yeast Mitochondrial Membrane Transporter Ctp1(p) in the Plasma Membrane of *Xenopus laevis* Oocytes.

**Fig. 1.**
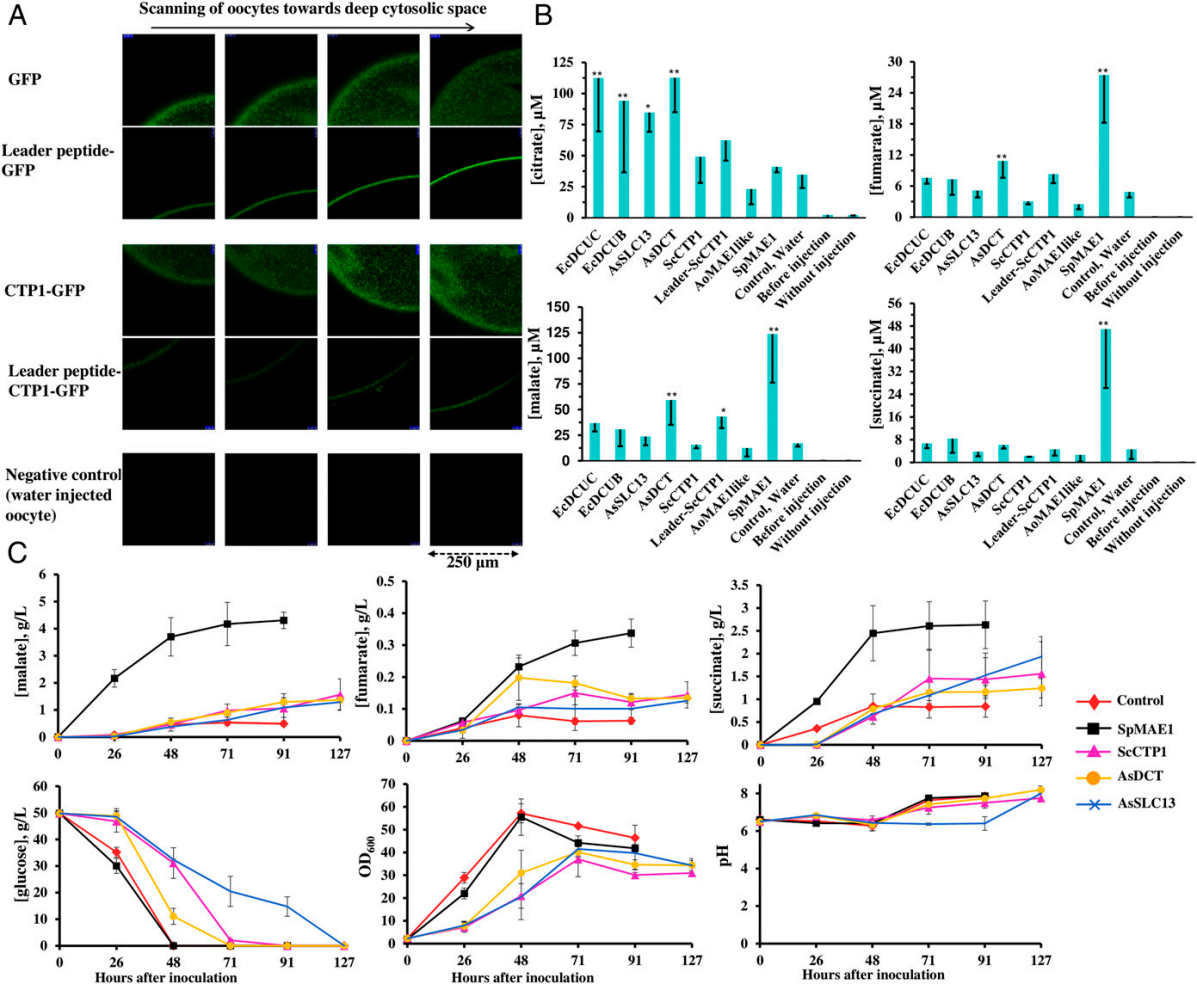
Carboxylic acid transport assays in *Xenopus* oocytes and *S. cerevisiae*. (*A*) The expression of GFP, *Sc*Ctp1-GFP, and their N-terminal fusions with the HsOrai1 leader peptide was examined by confocal microscopy, scanning oocytes on the *z* axis from the most outer surface toward the inner cytoplasmic space. (*B*) Efflux of carboxylic acids from oocytes. The bars represent the carboxylic acid contents of the medium (means of 3 to 4 biological replicates each involving 20 oocytes with SDs shown as error bars) 3 h after injecting fumarate and citrate into the control (water injected) oocytes with no heterologous transporter and into the oocytes expressing individual candidate transporters. Asterisks mark significant changes in comparison with the control (**P < 0.01, *P < 0.05). The carboxylic acid concentrations in the medium were also examined before metabolite injection (before injection) and after 3 h incubation of oocytes without injecting metabolites (without injection). (*C*) Time course of metabolite concentrations in the fermentation broth of the transporter-expressing *S. cerevisiae* strains and the control strain. Error bars show the SDs of 3 biological replicates.

We used the *Xenopus* oocytes for functional analysis of the transporters (24). To enable the study of a mitochondrial transporter, we designed a construct for targeting transporters into the plasma membrane. Here, we took advantage of the N-terminal segment of the human calcium release-activated calcium channel Orai1(p) (amino acids 71–246; GenBank: NP_116179). The N-terminal segment is responsible for protein localization in plasma membrane both in native cells and upon expression in *Xenopus* oocytes (26, 27). We combined the Orai1(p) peptide segment with Gfp(p) or with *Sc*Ctp1-Gfp as N-terminal fusions and expressed these constructs in oocytes. The localization was studied by confocal laser scanning microscopy, scanning along the *z* axis from the surface toward the deep cytosolic space of oocytes (27, 28). The *N*-tagged variants of Gfp(p) and Ctp1(p) were shown to localize in the plasma membrane of oocytes, while their nontagged variants were expressed in the cytosolic space (Fig. 1*A*).

### Functional Analysis of the Transporters in Oocytes.

The candidate membrane transporters (Table 1) were subjected to functional analysis upon expression in *Xenopus* oocytes. Oocytes expressing each of the candidate transporters were injected with citrate and fumarate (estimated internal concentrations of 2 and 1.5 mM, respectively), which could also be converted into succinate and malate through the TCA cycle within oocytes. After incubating the oocytes in a buffer for 3 h, the concentrations of exported dicarboxylic acid were measured by LC-MS. *Ec*Dcuc(p), *Ec*Dcub(p), *As*Slc13(p), and *As*Dct(p) were able to export citrate (Fig. 1*B*). *Sp*Mae1(p), the most closely related transporter to the Dcu(p) transporters did not show citrate export capability (Fig. 1*B*). On the other hand, *Sp*Mae1(p) was able to export fumarate, succinate, and malate (Fig. 1*B*). *As*Dct(p) and *Sc*Ctp1(p) fused to the Orai11(p) peptide (Leader-*Sc*Ctp1) showed some detectable export of fumarate and malate.

### Effect of the Transporters on the Production of C4-Dicarboxylic Acids in *S. cerevisiae*.

To examine the performance of the carboxylic acid transporters in a yeast cell factory, we expressed them in a *S. cerevisiae* strain engineered for the production of C4-dicarboxylic acids. The strain was created based on the evolved pyruvate decarboxylase-deficient strain (29) in which we overexpressed the native cytosolic pyruvate carboxylases Pyc1(p) and Pyc2(p) and malate dehydrogenase without the peroxisomal targeting signal (Mdh3[p]ΔSKL) to retain it in the cytosol (30). This engineered strain was able to produce up to 0.5 and 0.8 g/L of extracellular malate and succinate, respectively (Fig. 1*C*). To enhance the efflux, we additionally expressed the most efficient transporters determined in the *Xenopus* oocyte screen: *Sp*Mae1(p), *Sc*Ctp1(p), *As*Dct(p), and *As*Slc13(p) (Fig. 1*B*). In agreement with the oocyte assays, it was again the *Sp*Mae1(p) showing the highest transport rate (Fig. 1*C*). We found up to an 8-fold increase (4.3 g/L) in the malate titer upon expression of *Sp*Mae1 (Fig. 1*C*). The titer of succinate and fumarate also increased to 2.6 g/L (3-fold increase) and 0.33 g/L (5-fold increase), respectively (Fig. 1*C*). As expected, expression of *Sc*Ctp1(p), *As*Dct(p), and *As*Slc13(p) affected the growth of yeast, resulting in a lower final OD_600_ and a slower glucose utilization. On the contrary, the expression of *Sp*Mae1(p) did not exhibit a negative effect on the growth of yeast cells, and the glucose consumption was similar to the control strain without a heterologous transporter (Fig. 1*C*).

### *Sp*Mae1(p) Is a Member of the SLAC1 Family.

**Fig. 2.**
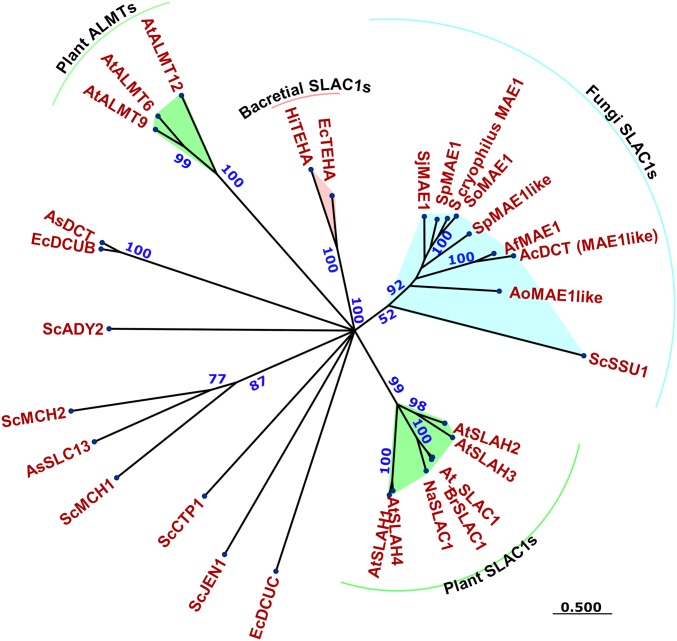
*Sp*Mae1(p) harbors a SLAC1 protein domain responsible for voltage-dependent transport. The maximum likelihood phylogenetic tree was built on the Whelan and Goldman substitutional matrix. Bootstraps and branch lengths are shown in blue and green, respectively. Protein domains were predicted with e values below 10^−40^. Sequence accession numbers are provided in *SI Appendix*, Table S6.

**Fig. 3.**
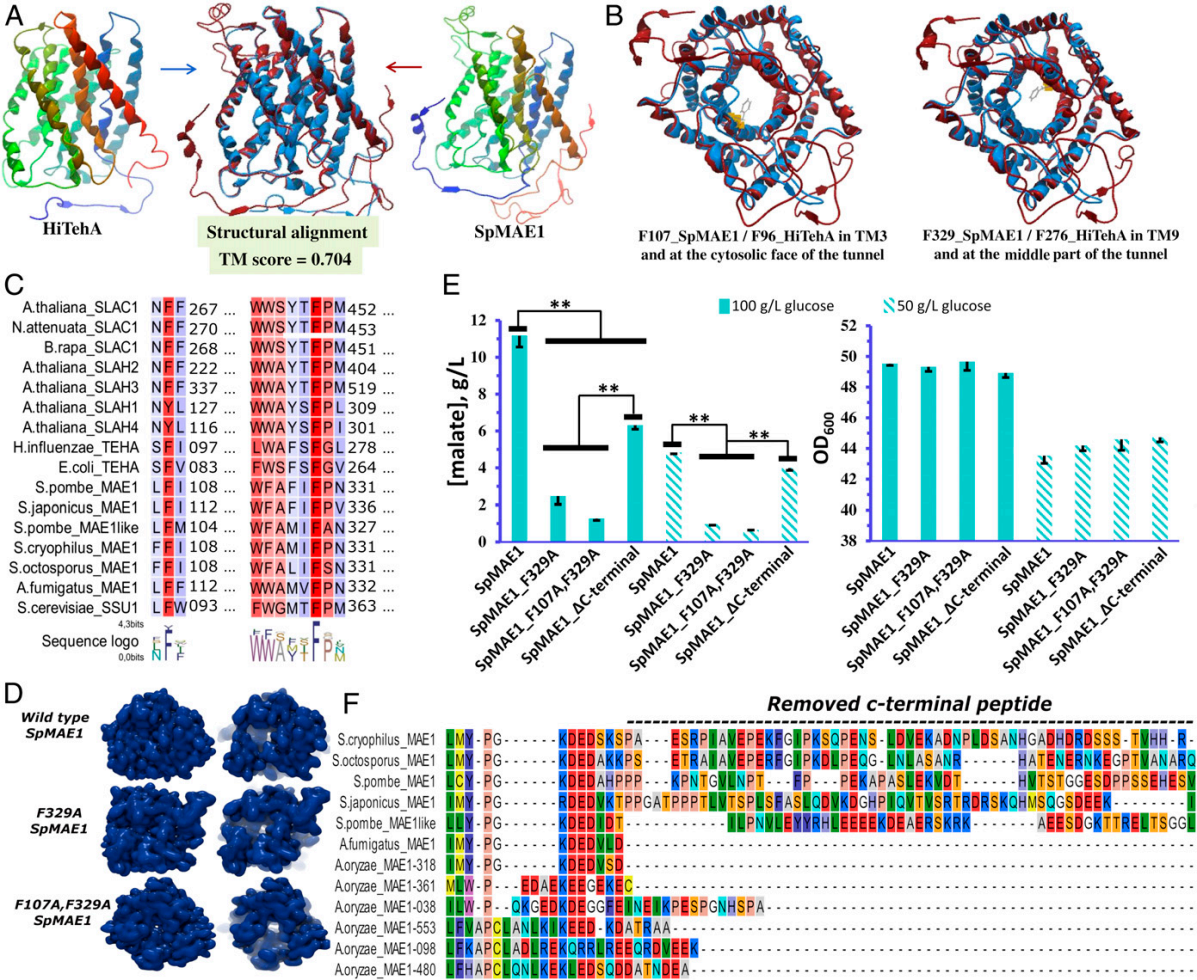
Structural homology of SLAC1 transporters and functional importance of the conserved regions. (*A*) Structural homology between *Hi*TehA(p) and *Sp*Mae1(p). (*B*) Cytosolic face representation of the *Hi*TehA(p) and *Sp*Mae1(p) transport tunnels with conserved phenylalanine residues blocking the substrate transport channel in SLAC1 transporters. (*C*) The 2 phenylalanine residues residing with their phenyl ring inside the transport channel (F107_*Sp*Mae1/F96_*Hi*TehA[p] and F329_*Sp*Mae1/F276_*Hi*TehA[p]) are conserved among the SLAC1 transporters. (*D*) Structural changes in *Sp*Mae1 after replacement of the conserved phenylalanines with alanine. (*E*) Malate concentration in the fermentation broth of yeast expressing wild-type *Sp*Mae1(p), *Sp*Mae1(p)^F329A^, *Sp*Mae1(p)^F107A,^ ^F329A^, and *SpMae*1(p)^∆C^ ^terminus^. Yeast strains were grown with 50 or 100 g/L of initial glucose concentration. Error bars represent SDs of 3 replicates. Statistical significant changes (*P* < 0.01) are highlighted by double asterisks. (*F*) The C-terminal differences of *Sp*Mae1(p) and its fungal homologs.

The *Sp*Mae1(p) transporter was initially identified on the basis of a mutant defective in malate uptake (19, 21, 31, 32). However, other experiments have also shown *Sp*Mae1(p)’s ability to improve the export of carboxylic acids, including malate from yeast cell factories (22, 23). In our experiments, the expression of *Sp*Mae1(p) did not impair the growth of yeast cells, while increasing the secretion of acids several fold. This was an indication that the production of acids was not coupled to growth, e.g., due to improved redox balance. The other transporters that also increased the secretion of acids all inhibited the cellular growth. We, therefore, hypothesized that the *Sp*Mae1(p) transporter must have a different transport mechanism with less energetic expenditure. To investigate this, we performed a protein motif search on *Sp*Mae1(p) using the Pfam library version 31 with 16,712 models (available at ftp://ftp.ebi.ac.uk/pub/databases/Pfam/releases/) and Gene3d models version 16 including 65,016 models (available at http://download.cathdb.info/gene3d/v16.0.0/gene3d_hmmsearch/). *Sp*Mae1(p) was annotated as a voltage-dependent slow activating (S-type) anion channel 1 (SLAC1) with e values below 10^−72^ (Fig. 2). The closest homolog of *Sp*Mae1(p) in *S. cerevisiae* is sulfite pump *Sc*Ssu1(p), and it contains a SLAC1 Pfam motif. The phylogenic relationship with other annotated Slac1(p) transporters from plants (33), bacteria (34), and with the closest homologs is illustrated in Fig. 2. The analysis also distinguished the aluminum-activated malate transporters (ALMTs) (Fig. 2), which are also voltage dependent (35, 36). It must be noted that malate transport by *Sp*Mae1(p) did not show any proton exchange in a previous study (21), which is in agreement with the SLAC1 family annotation. The predicted structure of *Sp*Mae1(p) is more similar (TM-score 0.704) to the experimentally determined structure of *Haemophilus influenza Hi*TehA(p) (34) as a bacterial homolog of plant Slac1(p) from *Arabidopsis thaliana* than to the transporters from the DCU and ALMT families (Fig. 3*A* and *SI Appendix*, Fig. S2).

### Conserved Phenylalanine Residue Found in the Transport Channel of Slac1(p) Transporters Is Critical for the Activity of *Sp*Mae1(p).

**Fig. 4.**
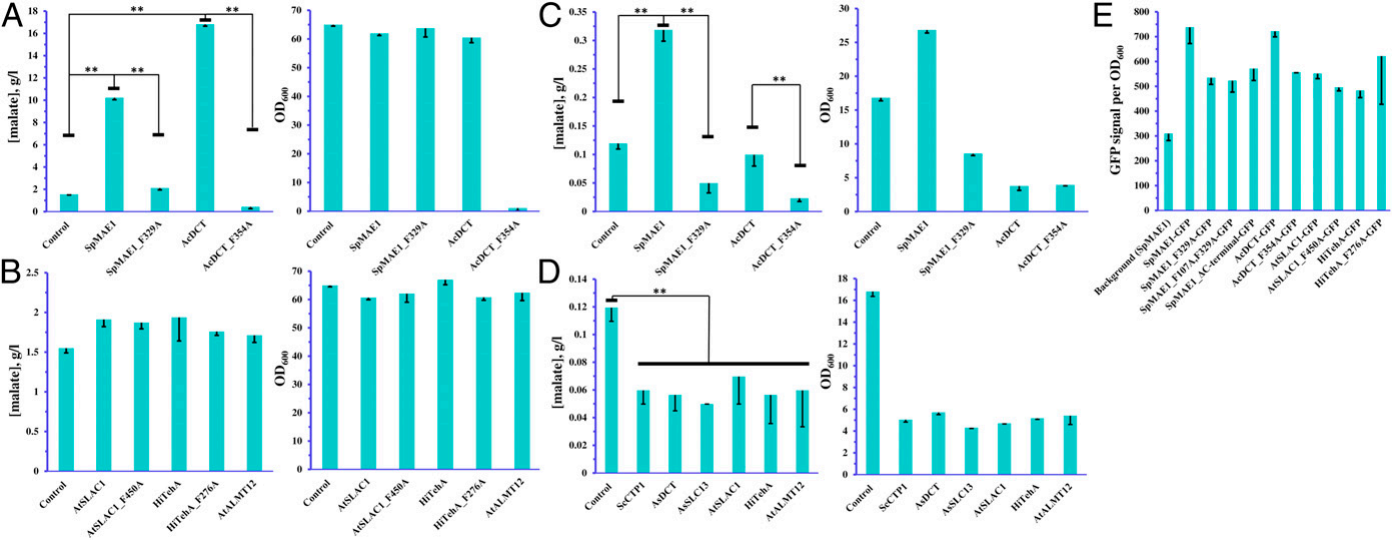
Effect of expression of native and F/A mutated transporter variants in yeast on the production of malate and on growth. (*A* and *B*) Malate titers in a mineral medium with calcium carbonate for buffering. (*C* and *D*) Malic acid titers in a mineral medium without pH buffering. (*E*) Normalized fluorescence of yeast cells expressing native and F/A mutated transporter variants fused with GFP at the C terminus. (*A*–*E*) Error bars represent SDs of 3 biological replicates. Statistical significant changes (*P* < 0.01) are highlighted by double asterisks.

Alignment of Slac1(p) transporters, including Mae1(p) transporters, uncovered 2 conserved phenylalanine residues located within the transport channel (F107_*Sp*Mae1[p] in transmembrane domain 3 and F329_*Sp*Mae1[p] in transmembrane domain 9). These residues close the transporter tunnel with their phenyl rings (Fig. 3 *B* and *C*). While the F329 of *Sp*Mae1(p) is 100% conserved in all of the investigated SLAC1 transporters, the F107 was substituted by similar amino acid tyrosine in *At*Slah1(p) and *At*Slah4(p) (Fig. 3*C*). To examine the essentiality of these residues, we created a single-residue mutant *Sp*Mae1(p)^F329A^ and a double-residue mutant *Sp*Mae1(p)^F107A,^ ^F329A^ and expressed them individually in *S. cerevisiae*. These changes removed the phenyl rings, respectively, from the middle or middle and cytosolic faces of the transport channel (Fig. 3*B*). While the *in silico* structure modeling predicted that mutations, particularly in combination, would widen the channel (Fig. 3*D*), the single mutation of F329A and the double mutation of F107A and F329A abolished the effect of *Sp*Mae1(p) on malate secretion (Fig. 3*E*). We additionally found 2 groups of Mae1(p) transporter by comparing Mae1(p) transporters of the *Aspergillus* species with the *Schizosaccharomyces* species, the latter distinguished by an extended C-terminal peptide (Fig. 3*F*). To examine the possible role of this C-terminal peptide, we removed the last 46 amino acids from *Sp*Mae1(p) (Fig. 3*F*) and found up to a 40% decrease in malate secretion from *S. cerevisiae* (Fig. 3*E*). This decrease could, however, be explained by the lower expression level of *Sp*Mae1(p) without the C-terminal peptide, which was 40% lower than the native protein. The expression levels were determined using C-terminal fusion with a GFP protein (Fig. 4*E*).

### Fungal Mae1 Transporter from *A. carbonarius* Also Increases Malate Secretion at Neutral pH.

We then examined the effect of several other SLAC1 transporters on malate production in yeasts. We selected *At*Slac1(p) from plant *A. thaliana*, *Hi*TehA(p) from bacterium *H. influenza*, and *Ac*Dct(p) from fungus *A. carbonarius*. We also included an ALMT member, *At*Almt12(p) from *A. thaliana*. Among the examined transporters, *Ac*Dct(p) expression resulted in a 12-fold increase in malate titer, while the rest of the transporters lead to a smaller increase in 10–20% (Fig. 4 *A* and *B*). These experiments were performed using calcium carbonate as the buffering agent in the medium as in the experiments described in the previous sections. To investigate the effect of transporters under low pH, we also performed the same experiment, now omitting the calcium carbonate from the medium. The initial pH of the medium was 4.8, and it rapidly declined to 2.4–2.6 during the cultivation. Overall, malic acid production and the growth were lower in the low-pH cultivation (Fig. 4 *C* and *D*). *Sp*Mae1(p) increased malic acid titer 3-fold and improved the growth (Fig. 4*C*), but all of the other transporters had a negative effect on both the malic acid titer and the growth.

For the SLAC1 members, we also examined the effect of mutations corresponding to the *Sp*Mae1(p)^F329A^. There were no significant changes in the activities of *At*Slac1(p) or *Hi*TehA(p). Expression of the mutant *Ac*Dct(p)^F354A^ resulted in a lower titer of malic acid than in the control strain not expressing a heterologous transporter at low pH (Fig. 4*C*). Curiously, the strain expressing *Ac*Dct(p)^F354A^ had a severe growth defect at neutral pH (Fig. 4*A*), so we could not draw conclusions about the activity of the mutated transporter at neutral pH. To ensure that the observed effects were not just due to the different expression levels of the mutated transporters, we expressed C-terminal GFP fusions of transporters in yeast and measured the fluorescence (Fig. 4*E* and *SI Appendix*, Fig. S3). The GFP signal for *Sp*Mae1(p) and *Ac*Dct(p) mutants was 40–50% lower than for the native transporters. The decreased expression of *Sp*Mae1(p) cannot explain the complete loss of activity by the mutated variant of *Sp*Mae1(p), so we can conclude that phenylalanine residue F329 is essential for the transporter activity.

## Discussion

Dicarboxylic acids, currently mainly produced from petroleum and gas, can be alternatively produced by fermentation of renewable feedstocks. Yeast cell factories are particularly attractive for these processes due to low-pH tolerance (37, 38). Carboxylates need membrane transporters to be secreted out of the cells (39, 40). Proton dissociation from carboxylic acids at neutral pH conditions releases membrane-nondiffusible anion carboxylates (37). The engineering of yeasts for malate production on carbon feedstock resulted in up to 70% of the maximum theoretical yield, and the malate was secreted into the fermentation medium (37, 41–43). Unlike bacterial succinic acid, producers that prefer neutral pH, *S. cerevisiae* can grow in an acidic medium with a pH range of 3–6, which reduces the need for neutralization and allows direct recovery of an undissociated form of acids (37). Channels, active pumps, permeases, and mitochondrial carriers are involved in the transport of carboxylic acids across the *S. cerevisiae* membranes (44). Improvement of malate, succinate, and fumarate secretion in yeast was obtained by expression of the malate transporter gene Mae1 from the fission yeast *S. pombe* (21, 22, 32, 45). Recently, *Ac*Dct(p), the homolog of *Sp*Mae1(p), was found to boost C4-dicarboxylic acid production in *A. carbonarius* (46). Originally, *Sp*Mae1(p) was annotated as a member of the TDT family and was believed to use a proton as the motive force (19). In agreement with the previous studies, we found that *Sp*Mae1(p) is highly active for the export of malate, succinate, and fumarate in oocytes (Fig. 1*B*) and in yeast cells (Fig. 1*C*). It was surprising that expression of *Sp*Mae1(p) did not affect the cellular growth, in contrast to *Sc*Ctp1(p), *As*Dct(p), and *As*Slc13(p) (Fig. 1*C*). Our phylogenetic and protein motif analyses annotated *Sp*Mae1(p) and *Ac*Dct(p) as members of the voltage-dependent Slac1(p) transporters (Fig. 2). Together with the rapidly activated Almt(p) channels (Fig. 2), Slac1(p) transporters respond to the voltage changes (depolarization) and export osmolytes, such as malate, nitrate, and chloride anions, which lead to stomatal closure in plants (33, 36, 47). Therefore, *Sp*Mae1(p) and *Ac*Dct(p) are most likely equipped with mechanisms used by their evolutionary and structurally closely related transporters of the SLAC1 family (Table 1, Fig. 2, and *SI Appendix*, Fig. S2). This is in contrast to the TDT family where the activity of transporters is coupled with a proton or Na^+^ ions. We recently addressed the energetic evolution of transporters, both at the level of cellular transportome and also transporter family levels (48). It may be that the same energetic evolution has been playing a role within the proton motive force driven TDT family, giving rise to the voltage-dependent transporters.

Unraveling the transport mechanism of *Sp*Mae1(p) has the potential for further improvements via engineering for higher transport efficiencies. There are two highly conserved phenylalanine residues in *Sp*Mae1(p) (Fig. 3). One of these residues has a phenyl ring at the cytosolic face of the transport pore and the other within the pore (Fig. 3 *B* and *C*). Replacing the inner phenylalanine with alanine in plant Slac1(p) and bacterial TehA(p) homologs has been shown to increase the chloride ion currents (34), which is in agreement with the structural changes, i.e., movements of the helices and widening of the channel, that we found should also happen in *Sp*Mae1(p) (Fig. 3*D*). However, our data indicate that these phenylalanine residues of F107 and F329 in *Sp*Mae1(p), while closing the transport channel in the substrate-free state, are also necessary for the transport of carboxylic acids (Figs. 3*E* and 4 *A* and *C*). As a conserved motif, the phenylalanine F107 and the flanking amino acids have notably been reported as a part of the substrate binding pocket in plant Slac1(p) transporters (49).

To summarize, we showed that the Mae1(p) transporter from *S. pombe* had a very high activity toward C4-dicarboxylic acids (succinic, malic, and fumaric) in both *Xenopus* oocytes and yeast *S. cerevisiae* and that *Sp*Mae1(p) did not inhibit the growth of yeast cells both at neutral and at low pH. A homolog *Ac*Dct(p) from *A. carbonarius* could also increase the production of malate in yeast without inhibiting the growth, albeit only at neutral pH. We present evidence that *Sp*Mae1(p) and *Ac*Dct(p) belong to the voltage-gated anion channel family SLAC1 and their expression results in energetically efficient export of dicarboxylic acids. This finding is important for engineering efficient cell factories for the production of biobased organic acids.

## Materials and Methods

All of the DNA constructs were built using the USER fusion technique. In vitro transcribed cRNAs were injected into the *X. laevis* oocytes by RoboInject (Multi Channel Systems, Germany). Candidates of membrane transporters were expressed in the *S. cerevisiae* cell factory designed to produce malic acid. A Leica TCS SP5-II confocal microscope was used for localization studies. HMMER version 3.1b2 and Phyre2 were used for protein motif identification and structure predictions, respectively. Chemicals were quantified by HPLC and LC-MS. Detailed experimental procedures can be found in *SI Appendix*, *Supplementary Materials and Methods* and Tables S1–S5.

## Supplementary Material

Supplementary File
