# Supplementary material for: Engineering energetically efficient transport of dicarboxylic acids in yeast Saccharomyces cerevisiae
Source: Proc Natl Acad Sci U S A. 2019 Aug 29;116(39):19415–20. doi: 10.1073/pnas.1900287116 (PMC6765260; doi:10.1073/pnas.1900287116)
Supplement: Supplementary File [file pnas.1900287116.sapp.pdf]

PNAS
www.pnas.org

Supplementary Information for

Engineering energetically efficient transport of dicarboxylic acids in yeast *Saccharomyces cerevisiae*

Behrooz Darbani, Vratislav Stovicek, Steven Axel van der Hoek, Irina Borodina

Irina Borodina
Email: irbo@biosustain.dtu.dk

This PDF file includes:

Supplementary Materials and Methods
Figures S1 and S3
Tables S1 to S6
References for SI reference citations

www.pnas.org/cgi/doi/10.1073/pnas.1900287116

Supplementary Materials and Methods

Media and yeast cultivation conditions

Yeast cells were grown in standard YPD medium at 30°C. For selection, SC plates (20 g/L agar) without leucine, uracil or histidine or a combination of these were used. Production of dicarboxylic acids was demonstrated in 250 ml baffled shake flasks and 50-ml working volume of a defined mineral medium with 50 g/L glucose unless indicated. The agitation was 250-rpm. The mineral medium consisted of 1.5 g/L urea, 3 g/L KH_2PO_4, 6.6 g/L K_2SO_4, 0.5 g/L $MgSO_4 \cdot 7H_2O$, 1 ml/l of vitamin solution and 2 ml/l trace elements as described in (1) and pH was set to 4.8. Cells from the seed culture were washed with sterile water, resuspended in the medium and added to deep-well plates or shake flasks containing $CaCO_3$ (2) in a final concentration of 25 g/L. The cells were inoculated to initial OD_{600} of 2 (ca. 0.3 g/L cell dry weight). For seed culture medium, 7.5 g/L $(NH_4)_2SO_4$ was used instead of urea and K_2SO_4 was omitted. Further, 14.4 g/L KH_2PO_4 and 20 g/L glucose was used and pH set to 6.

Strain construction

All yeast strains constructed are derived from CEN.PK TAM strain (3) and listed in Table S1. Native and heterologous genes under the control of strong constitutive promoters were integrated into the genome of the parental yeast strain. Before yeast transformation, the integrative vectors were linearized by FastDigest *NotI* (ThermoFisher Scientific) restriction enzyme. Yeast cells were transformed by the standard PEG/LiAc method (4). The cells were plated on selective plates with the appropriate selection. The plates were typically incubated for 3-5 days. Verification of correct integrations was done by colony PCR using OneTaq® Hot Start Quick-Load® 2X Master Mix (New England Biolabs) using the manufacturer's protocol and primers listed in Table S2.

DNA Constructs

The integrative plasmids (Table S3) were constructed by USER fusion (5). The particular gene and promoter BioBricks (Table S4) were amplified by PCR with Phusion U polymerase (ThermoFisher Scientific). Used primers and templates are listed in Table S2 and Table S4. Native genes were amplified from CEN.PK genomic DNA. Heterologous genes were synthetized by GeneArt. The exception was *Sp*Mae1, which was amplified from *S. pombe* genomic DNA. Empty integrative vectors were digested with FastDigest *SfaAI* (ThermoFisher Scientific) restriction endonuclease, nicked with *Nb.BsmI* (New England BioLabs) and assembled with a PCR amplified gene and a promoter of choice. To express the transporter coding genes in oocytes, genes were cloned downstream of the T7 promoter in the USER compatible *Xenopus* expression vector pUSER016 (6). The empty vector was digested by PacI and *Nt.BbvCI* (New England BioLabs). The amplified DNA fragments were gel purified and together with the linearized plasmids incubated with USER enzyme (New England BioLabs) for 25 min at 37°C, followed by incubation at 25°C for 25 min. The reactions were transformed into chemically competent *E. coli* cells. All the cloned plasmids were verified by Sanger sequencing.

Transport assays in Xenopus oocytes

The *Xenopus laevis* oocytes were obtained from Ecocyte Bioscience (Germany) and kept at 18°C. Linear cassettes (including T7 promoter, the gene of interest, and 3UTR) were amplified with Phusion Hot Start polymerase (ThermoFisher Scientific) and used as template for *in-vitro* transcription. Capped cRNAs was synthesized using the mMESSAGE mMACHINE® T7 Transcription Kit (AM1344; ThermoFisher). The quality and quantity of RNAs were determined by Agilent 2100 Bioanalyzer. For expression in oocytes, 25 ng of *in-vitro* produced cRNAs for the transporters was microinjected into oocytes 3 days prior to transport assays (6). For microinjection of cRNAs and compounds, we used the RoboInject (Multi Channel Systems, Germany) automatic injection system (7, 8). Injection needles with opening of 25 µm were used (Multi Channel Systems). The stock solution of 50 nl containing 40 mM citrate and 30 mM fumarate was used for microinjection into the oocytes to obtain estimated internal concentrations of 2 mM and 1.5 mM, respectively, assuming an after-injection dilution factor of 20 (6). Following four washing steps, each batch of 20 oocytes was incubated for 180 min in 90 µl Kulori buffer at pH 5. After incubation, 70 µl of the medium was collected from each batch with intact oocytes and added onto 70 µl 60% MeOH before LC-MS analysis. Statistical significant differences were determined through one-way ANOVA followed by Duncan's Multiple Range Test.

Chemicals and HPLC/LC-MS analyses

Residual calcium carbonate in the cultivation broth was dissolved by adding HCl to a final concentration up to 0.5 M before the samples of fermentation broth were centrifuged. Glucose and other metabolites levels in the culture supernatants were determined by HPLC. Aminex HPX87H ion exclusion column (300x7.8 mm, 9µm) at 60°C with 5 mM H_2SO_4 as the mobile phase was used. Glucose, acetate and glycerol were detected by RI-detector. Succinic and fumaric acid were detected by UV detector at 205 nm. The data was acquired and analyzed with Chromeleon software. Malic acid quantification was performed spectrophotometrically using a malic acid assay kit (K-LMAL-58A; Megazyme). For oocyte transport assays, metabolite levels were measured using LC-MS. The LC-MS data was collected on EVOQ EliteTriple Quadrupole Mass Spectrometer system coupled with an Advance UHPLC pump (Bruker, Fremont Ca). Samples were held in the CTC HTS PAL autosampler at a temperature of 5.0 °C during the analysis. Injections of the samples with 1uL in volume were made onto a Waters ACQUITY HSS T3 C18 UHPLC column, with a 1.8 um particle size, 2.1 mm i.d. and 100 mm long. The column was held at 30.0 °C. The solvent consisted of solvent A (milliQ water with 0.1% formic acid) and solvent B (acetonitrile with 0.1% formic acid). The flow rate was 0.400 ml/min with an initial solvent composition of %A = 100, %B = 0 held until 0.50 min, the solvent composition was then changed following a linear gradient until it reached %A = 5.0 and %B = 95.0 at 1.00 min. This was held until 1.79 min when the solvent was returned to the initial conditions and the column was re-equilibrated until 4.00 min. The column eluent flowed directly into the Heated ESI probe of the MS which was held at 250 °C and a voltage of 2500 V. MRM data was collected in negative ion mode. The target masses are shown in Table S5. The other MS settings were as follows: Sheath Gas Flow Rate of 50 units, Nebuliser Gas Flow Rate of 50 units, Cone Gas Flow Rate of 20 units, collision gas pressure of 1 mTorr, and Cone Temp at 350 °C.

Confocal microscopy and expression analysis

Transient expression in oocytes was performed by injecting cRNAs into the oocytes. Visualization of the GFP and its fusions derivatives in *Xenopus* oocytes was carried out using a Leica TCS SP5-II confocal microscope. To minimize autofluorescence of oocytes, excitation was performed with a low intensity of 16% at 488 nm and using Argon-ion laser at 20% intensity. Emission was also limited to the narrow range of 504-515 nm. GFP fused to the C-terminal of the wild-type and F/A mutated transporters were used to detect GFP signal of strains using microtiter plate reader (excitation was at 485 nm and emission at 515 nm).

Structural and functional-motif analyses

Protein domain predictions were performed by HMMER version 3.1b2 (9). This was done based on an in-house indexed target population of 16712 protein superfamily domains, which was built from Pfam library version 31 (available at ftp://ftp.ebi.ac.uk/pub/databases/Pfam/releases/) and also 65016 motif-domains from Gene3d version 16 (http://download.cathdb.info/gene3d/v16.0.0/gene3d_hmmsearch/). Transmembrane domains were predicted by TMHMM server v. 2.0 (10) and visualized by TMRPres2D (11). Three dimensional structures of *Hi*TehA(p) and *Sp*Mae1(p) were built using the Phyre2 (12) and based on the crystal structure of *Hi*TehA(p) (13). Structural alignment was performed by CLC Main Workbench version 7.6.4 (QIAGEN, Aarhus A/S). The degree of structural accordance quantified as TM score (14), a tradeoff between the alignment length and the alignment accuracy ranged from 0 to 1 (1.0 for identical proteins, > 0.7 for highly similar models, and < 0.5 for different folds). The maximum likelihood phylogenetic trees were built on the WAG substitutional matrix-based model for amino acid sequences and a bootstrap value of 1000 (15)

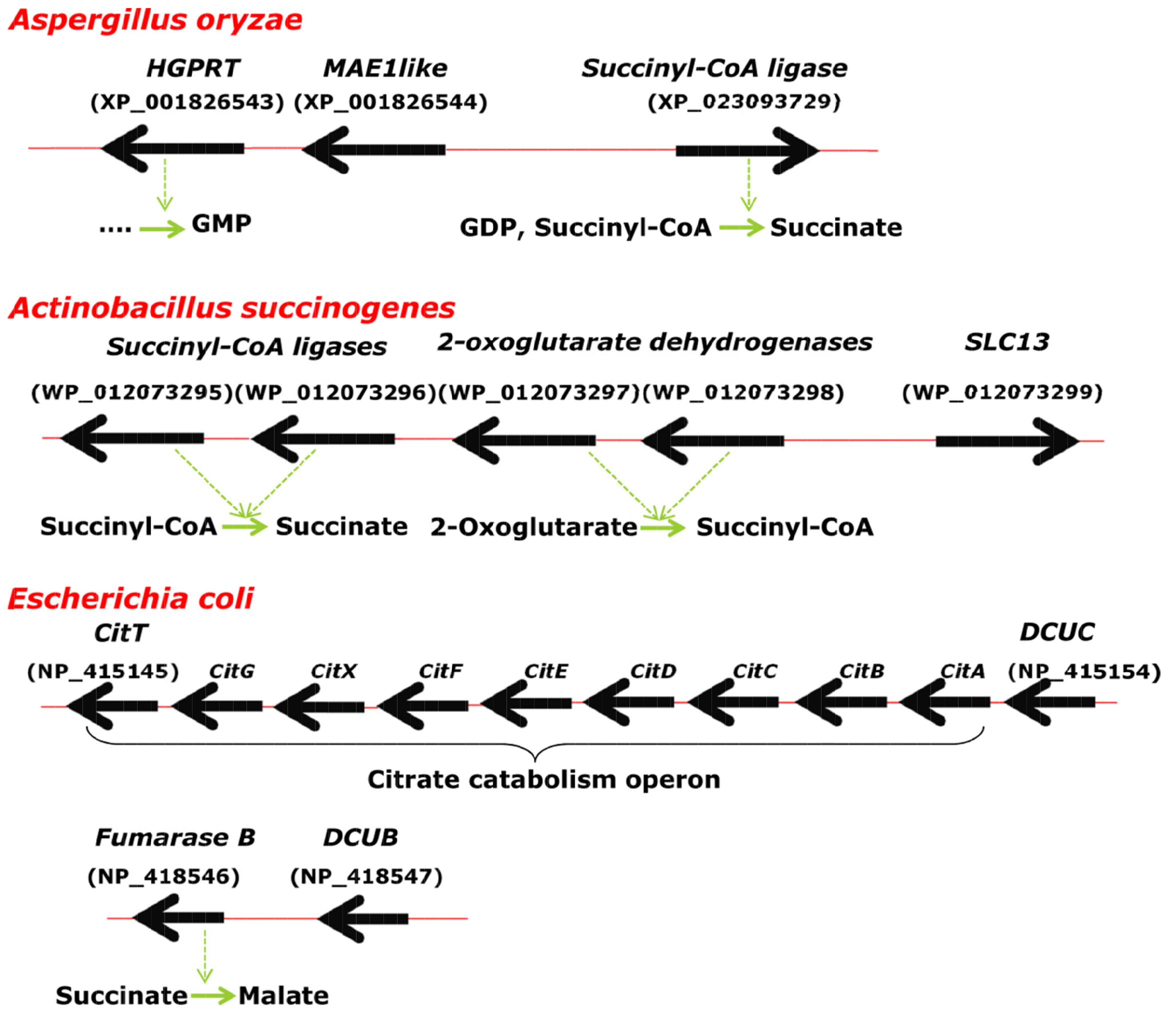

Fig. S1: Identified gene clusters with candidate genes coding for transporters

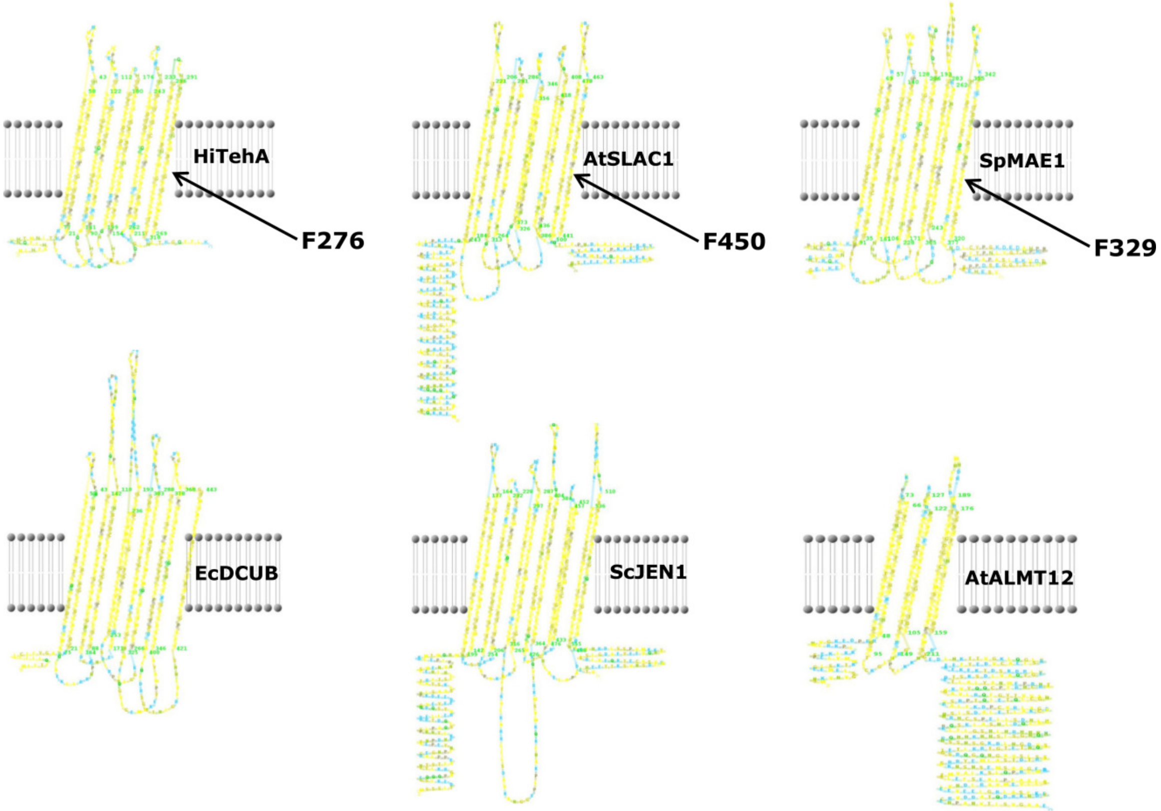

Fig. S2. Similarity of the transmembrane domains within the SLAC1 family transporters and their differences with other carboxylic acid transporters.

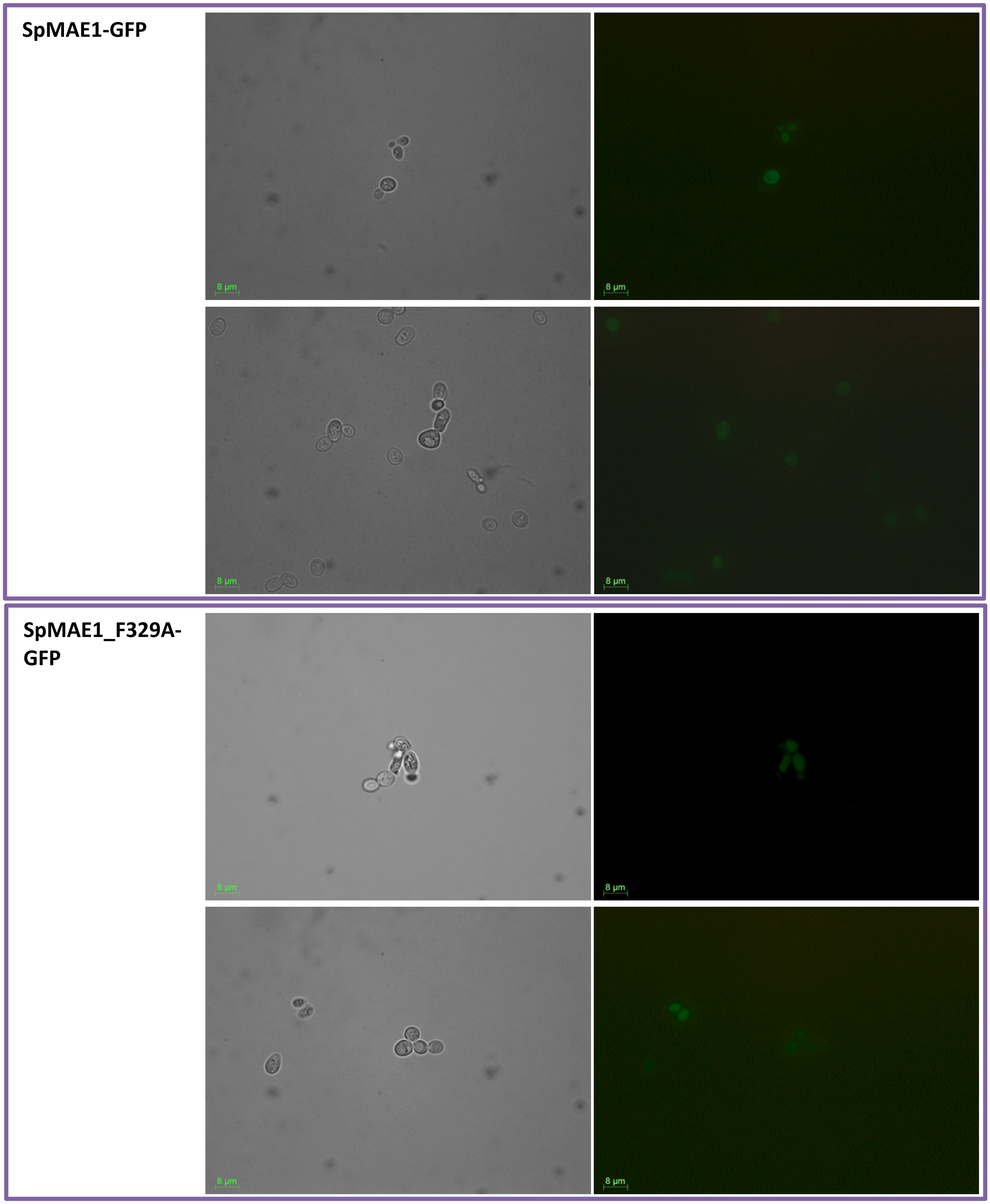

Fig. S3**: The expression of GFP fusions of wild-type and mutated transporters in yeast.**

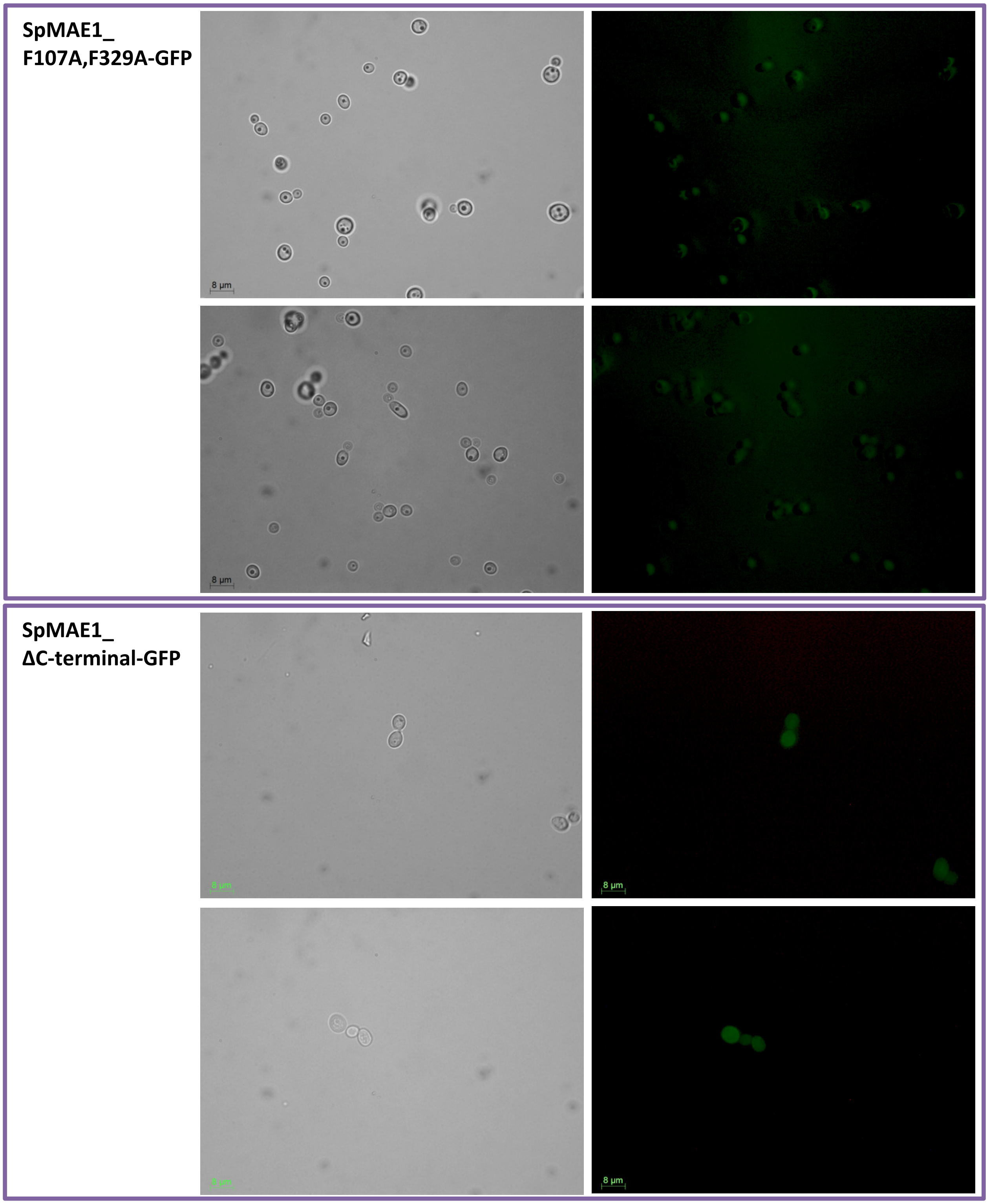

Fig. S3**: The expression of GFP fusions of wild-type and mutated transporters in yeast.**

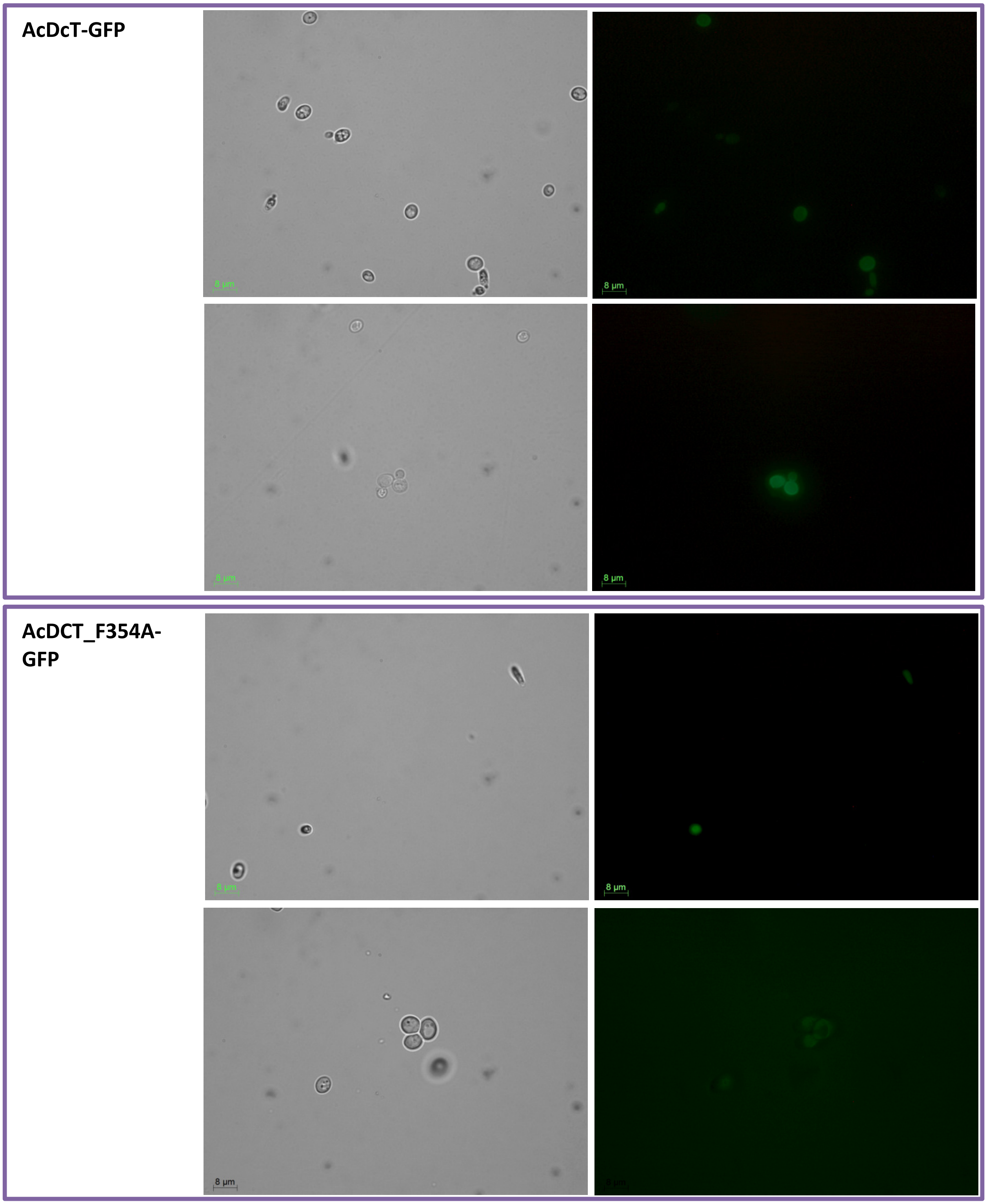

Fig. S3**: The expression of GFP fusions of wild-type and mutated transporters in yeast.**

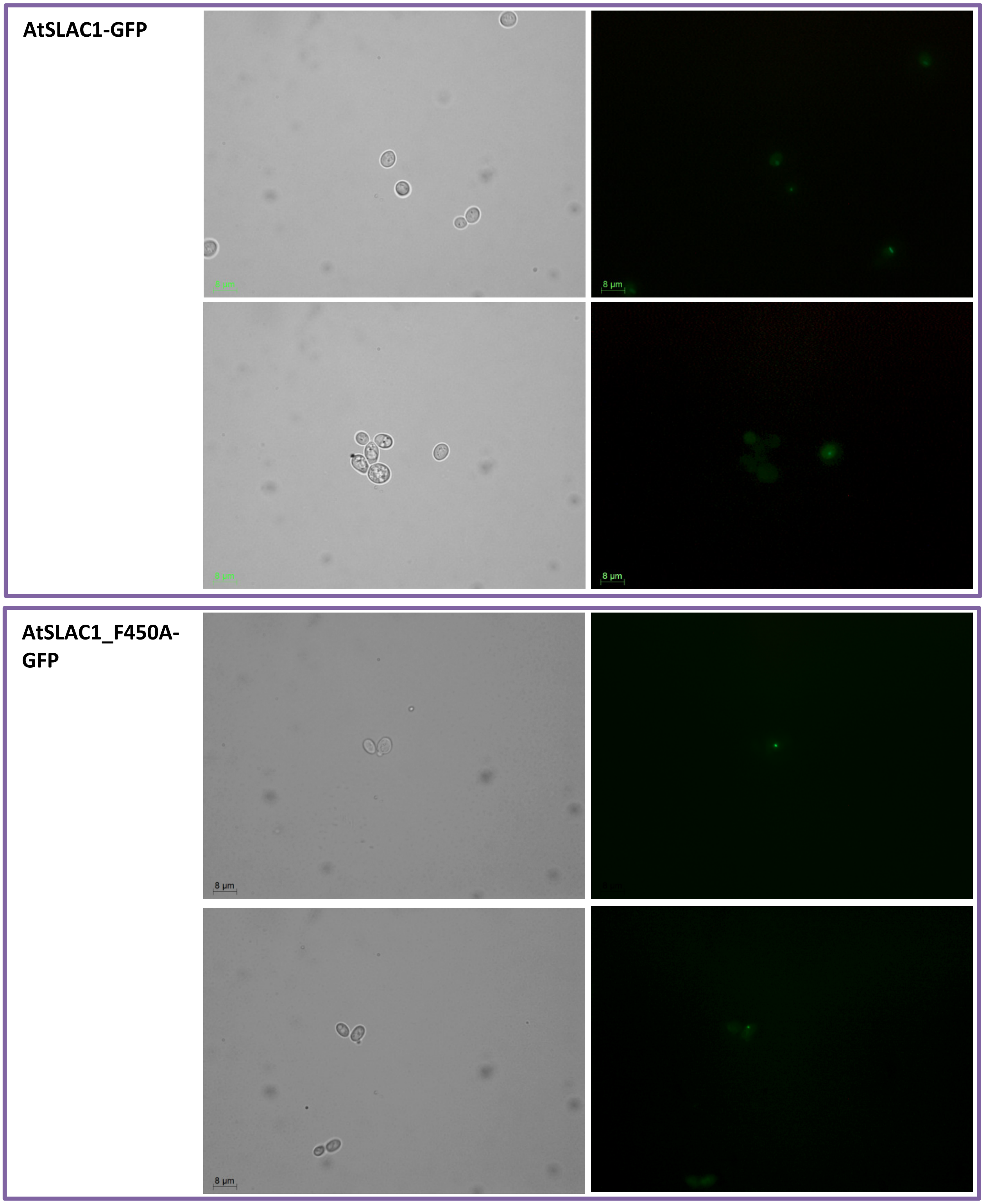

Fig. S3**: The expression of GFP fusions of wild-type and mutated transporters in yeast.**

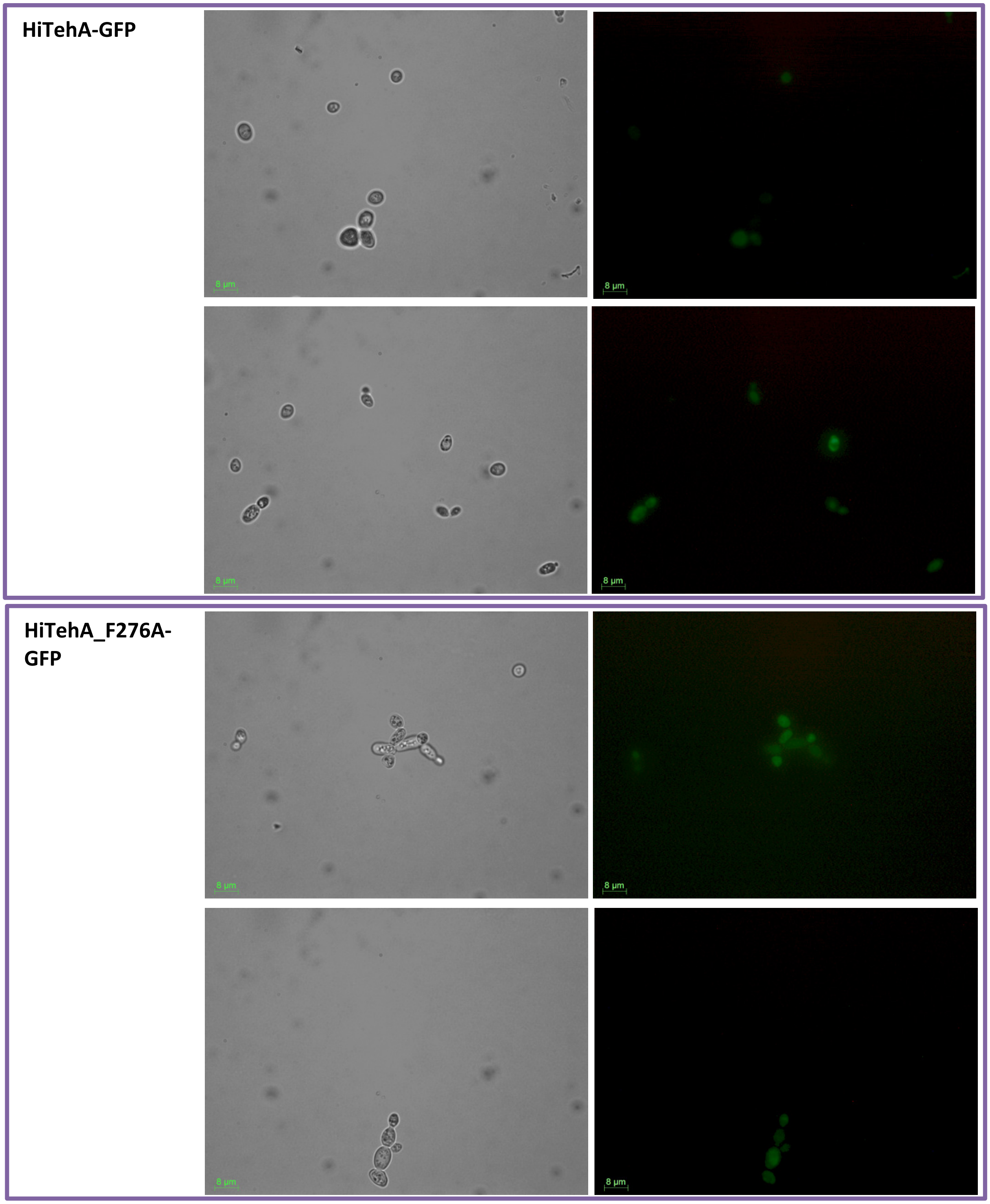

Fig. S3**: The expression of GFP fusions of wild-type and mutated transporters in yeast.**

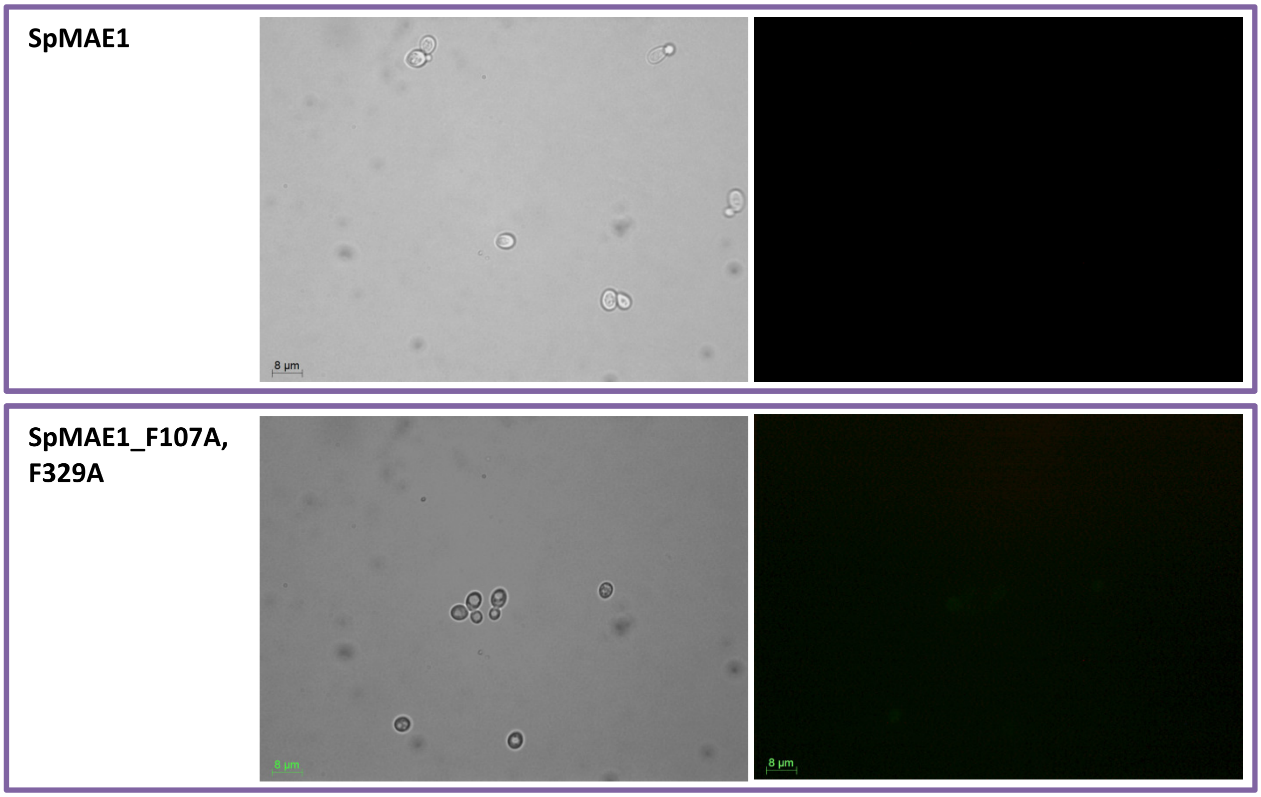

Fig. S3**: The expression of GFP fusions of wild-type and mutated transporters in yeast.**

Table S1. List of *S. cerevisiae* strains.

Strain name	ID	Genotype	Integrated plasmids
ST2681 (TAM ura-his-leu-)	2681	*MATa ura, his, leu, pdc1, pdc5, pdc6*	-
ST2757 (TAM+PYC1-PYC2)	2757	*MATa ura, his, pdc1, pdc5, pdc6, TEF1p-PYC1, PGK1p-PYC2, KlLEU2*	p0743 (pXI-1-loxP-KlLEU2-PYC1<-PTEF1-PPGK1->PYC2)
CEN.PK PYC1/PYC2/ScMDH3deltaSKL	6353	*MATa his, pdc1, pdc5, pdc6, TEF1p-PYC1, PGK1p-PYC2, KlLEU2, TDH3p-MDH3Δskl, KlURA3*	p0743 (pXI-1-loxP-KlLEU2-PYC1<-PTEF1-PPGK1->PYC2), p2860 (pX-2-loxP-KlURA-ScMdh3deltaSKL<-PTDH3)
CEN.PK PYC1/PYC2/ScMDH3deltaSKL	6438	*MATa pdc1, pdc5, pdc6, TEF1p-PYC1, PGK1p-PYC2, KlLEU2, TDH3p-MDH3Δskl, KlURA3, SpHIS5*	p0743 (pXI-1-loxP-KlLEU2-PYC1<-PTEF1-PPGK1->PYC2), p2860 (pX-2-loxP-KlURA-ScMdh3deltaSKL<-PTDH3), pCfB2228 (XII-4-loxP-SpHIS5syn)
CEN.PK MA_tr_SpMAE1	6442	*MATa pdc1, pdc5, pdc6, TEF1p-PYC1, PGK1p-PYC2, KlLEU2, TDH3p-MDH3Δskl, KlURA3, TEF1p-SpMAE1, SpHIS5*	p0743 (pXI-1-loxP-KlLEU2-PYC1<-PTEF1-PPGK1->PYC2), p2860 (pX-2-loxP-KlURA-ScMdh3deltaSKL<-PTDH3), pCfB6547 (XII-4-HIS5syn-pTEF1-SpMae1)
CEN.PK MA_tr_SpMAE1-GFP	9173	*MATa pdc1, pdc5, pdc6, TEF1p-PYC1, PGK1p-PYC2, KlLEU2, TDH3p-MDH3Δskl, KlURA3, TEF1p-SpMAE1-GFP, SpHIS5*	p0743 (pXI-1-loxP-KlLEU2-PYC1<-PTEF1-PPGK1->PYC2), p2860 (pX-2-loxP-KlURA-ScMdh3deltaSKL<-PTDH3), pCfB9031 (XII-4-HIS5syn-pTEF1-SpMae1-GFP)
CEN.PK MA_tr_ScCTP1	6444	*MATa pdc1, pdc5, pdc6, TEF1p-PYC1, PGK1p-PYC2, KlLEU2, TDH3p-MDH3Δskl, KlURA3, TEF1p-CTP1, SpHIS5*	p0743 (pXI-1-loxP-KlLEU2-PYC1<-PTEF1-PPGK1->PYC2), p2860 (pX-2-loxP-KlURA-ScMdh3deltaSKL<-PTDH3), pCfB6549 (XII-4-HIS5syn-pTEF1-ScCTP1)
CEN.PK MA_tr_ScCTP1leader	6445	*MATa pdc1, pdc5, pdc6, TEF1p-PYC1, PGK1p-PYC2, KlLEU2, TDH3p-MDH3Δskl, KlURA3, TEF1p-leaderCTP1, SpHIS5*	p0743 (pXI-1-loxP-KlLEU2-PYC1<-PTEF1-PPGK1->PYC2), p2860 (pX-2-loxP-KlURA-ScMdh3deltaSKL<-PTDH3), pCfB6550 (XII-4-HIS5syn-pTEF1-ScCTP1leader)
CEN.PK MA_tr_EcDCUC	6446	*MATa pdc1, pdc5, pdc6, TEF1p-PYC1, PGK1p-PYC2, KlLEU2, TDH3p-MDH3Δskl, KlURA3, TEF1p-EcDcuC, SpHIS5*	p0743 (pXI-1-loxP-KlLEU2-PYC1<-PTEF1-PPGK1->PYC2), p2860 (pX-2-loxP-KlURA-ScMdh3deltaSKL<-PTDH3), pCfB6551 (XII-4-HIS5syn-pTEF1-EcDcuC)
CEN.PK MA_tr_EcDCUB	6447	*MATa pdc1, pdc5, pdc6, TEF1p-PYC1, PGK1p-PYC2, KlLEU2, TDH3p-MDH3Δskl, KlURA3, TEF1p-EcDcuB, SpHIS5*	p0743 (pXI-1-loxP-KlLEU2-PYC1<-PTEF1-PPGK1->PYC2), p2860 (pX-2-loxP-KlURA-ScMdh3deltaSKL<-PTDH3), pCfB6552 (XII-4-HIS5syn-pTEF1-EcDcuB)
CEN.PK MA_tr_AsDCT	6448	*MATa pdc1, pdc5, pdc6, TEF1p-PYC1, PGK1p-PYC2, KlLEU2, TDH3p-MDH3Δskl, KlURA3, TEF1p-AsDcT, SpHIS5*	p0743 (pXI-1-loxP-KlLEU2-PYC1<-PTEF1-PPGK1->PYC2), p2860 (pX-2-loxP-KlURA-ScMdh3deltaSKL<-PTDH3), pCfB6553 (XII-4-HIS5syn-pTEF1-AsDcT)
CEN.PK MA_tr_AsSLC13	6449	*MATa pdc1, pdc5, pdc6, TEF1p-PYC1, PGK1p-PYC2, KlLEU2, TDH3p-MDH3Δskl, KlURA3, TEF1p-AsSlc13, SpHIS5*	p0743 (pXI-1-loxP-KlLEU2-PYC1<-PTEF1-PPGK1->PYC2), p2860 (pX-2-loxP-KlURA-ScMdh3deltaSKL<-PTDH3), pCfB6554 (XII-4-HIS5syn-pTEF1-AsSlc13)
CEN.PK MA_tr_AoMAE1like	6450	*MATa pdc1, pdc5, pdc6, TEF1p-PYC1, PGK1p-PYC2, KlLEU2, TDH3p-MDH3Δskl, KlURA3, TEF1p-AoMAE1like, SpHIS5*	p0743 (pXI-1-loxP-KlLEU2-PYC1<-PTEF1-PPGK1->PYC2), p2860 (pX-2-loxP-KlURA-ScMdh3deltaSKL<-PTDH3), pCfB6555 (XII-4-HIS5syn-pTEF1-AoMAE1like)
CEN.PK MA_tr_AcDCT (AcMAE1)	9159	*MATa pdc1, pdc5, pdc6, TEF1p-PYC1, PGK1p-PYC2, KlLEU2, TDH3p-MDH3Δskl, KlURA3, TEF1p-AcDCT, SpHIS5*	p0743 (pXI-1-loxP-KlLEU2-PYC1<-PTEF1-PPGK1->PYC2), p2860 (pX-2-loxP-KlURA-ScMdh3deltaSKL<-PTDH3), pCfB9017 (XII-4-HIS5syn-pTEF1-AcDCT)
CEN.PK MA_tr_ΔF354A_AcDCT (AcMAE1)	9163	*MATa pdc1, pdc5, pdc6, TEF1p-PYC1, PGK1p-PYC2, KlLEU2, TDH3p-MDH3Δskl, KlURA3, TEF1p--ΔF354A_AcDCT, SpHIS5*	p0743 (pXI-1-loxP-KlLEU2-PYC1<-PTEF1-PPGK1->PYC2), p2860 (pX-2-loxP-KlURA-ScMdh3deltaSKL<-PTDH3), pCfB9021 (XII-4-HIS5syn-pTEF1-ΔF354A_AcDCT)
CEN.PK MA_tr_AcDCT-GFP (AcMAE1-GFP)	9166	*MATa pdc1, pdc5, pdc6, TEF1p-PYC1, PGK1p-PYC2, KlLEU2, TDH3p-MDH3Δskl, KlURA3, TEF1p-AcDCT-GFP, SpHIS5*	p0743 (pXI-1-loxP-KlLEU2-PYC1<-PTEF1-PPGK1->PYC2), p2860 (pX-2-loxP-KlURA-ScMdh3deltaSKL<-PTDH3), pCfB9024 (XII-4-HIS5syn-pTEF1-AcDCT-GFP)
CEN.PK MA_tr_ΔF354A_AcDCT-GFP (AcMAE1-GFP)	9170	*MATa pdc1, pdc5, pdc6, TEF1p-PYC1, PGK1p-PYC2, KlLEU2, TDH3p-MDH3Δskl, KlURA3, TEF1p--ΔF354A_AcDCT-GFP, SpHIS5*	p0743 (pXI-1-loxP-KlLEU2-PYC1<-PTEF1-PPGK1->PYC2), p2860 (pX-2-loxP-KlURA-ScMdh3deltaSKL<-PTDH3), pCfB9028 (XII-4-HIS5syn-pTEF1-ΔF354A_AcDCT-GFP)
CEN.PK MA_tr_AtSLAC1	9161	*MATa pdc1, pdc5, pdc6, TEF1p-PYC1,*	p0743 (pXI-1-loxP-KlLEU2-PYC1<-PTEF1-

		PGK1p-PYC2, KlLEU2, TDH3p-MDH3Δskl, KlURA3, TEF1p-AtSLAC1, SpHIS5	PPGK1->PYC2), p2860 (pX-2-loxP-KlURA-ScMdh3deltaSKL<-PTDH3), pCfB9019 (XII-4-HIS5syn-pTEF1-AtSLAC1)
CEN.PK MA_tr_ΔF450A_ASLAC1	9164	*MATa pdc1, pdc5, pdc6, TEF1p-PYC1, PGK1p-PYC2, KlLEU2, TDH3p-MDH3Δskl, KlURA3, TEF1p--*ΔF450A_*AtSLAC1, SpHIS5*	p0743 (pXI-1-loxP-KlLEU2-PYC1<-PTEF1-PPGK1->PYC2), p2860 (pX-2-loxP-KlURA-ScMdh3deltaSKL<-PTDH3), pCfB9022 (XII-4-HIS5syn-pTEF1-ΔF450A_AtSLAC1)
CEN.PK MA_tr_AtSLAC1-GFP	9168	*MATa pdc1, pdc5, pdc6, TEF1p-PYC1, PGK1p-PYC2, KlLEU2, TDH3p-MDH3Δskl, KlURA3, TEF1p-AtSLAC1-GFP, SpHIS5*	p0743 (pXI-1-loxP-KlLEU2-PYC1<-PTEF1-PPGK1->PYC2), p2860 (pX-2-loxP-KlURA-ScMdh3deltaSKL<-PTDH3), pCfB9026 (XII-4-HIS5syn-pTEF1-AtSLAC1-GFP)
CEN.PK MA_tr_ΔF450A_ASLAC1-GFP	9171	*MATa pdc1, pdc5, pdc6, TEF1p-PYC1, PGK1p-PYC2, KlLEU2, TDH3p-MDH3Δskl, KlURA3, TEF1p--*ΔF450A_*AtSLAC1-gfp, SpHIS5*	p0743 (pXI-1-loxP-KlLEU2-PYC1<-PTEF1-PPGK1->PYC2), p2860 (pX-2-loxP-KlURA-ScMdh3deltaSKL<-PTDH3), pCfB9029 (XII-4-HIS5syn-pTEF1-ΔF450A_AtSLAC1-GFP)
CEN.PK MA_tr_HiTehA	9162	*MATa pdc1, pdc5, pdc6, TEF1p-PYC1, PGK1p-PYC2, KlLEU2, TDH3p-MDH3Δskl, KlURA3, TEF1p-HiTehA, SpHIS5*	p0743 (pXI-1-loxP-KlLEU2-PYC1<-PTEF1-PPGK1->PYC2), p2860 (pX-2-loxP-KlURA-ScMdh3deltaSKL<-PTDH3), pCfB9020 (XII-4-HIS5syn-pTEF1-HiTehA)
CEN.PK MA_tr_ΔF276A_HiTehA	9165	*MATa pdc1, pdc5, pdc6, TEF1p-PYC1, PGK1p-PYC2, KlLEU2, TDH3p-MDH3Δskl, KlURA3, TEF1p--*ΔF276A_*HiTehA, SpHIS5*	p0743 (pXI-1-loxP-KlLEU2-PYC1<-PTEF1-PPGK1->PYC2), p2860 (pX-2-loxP-KlURA-ScMdh3deltaSKL<-PTDH3), pCfB9023 (XII-4-HIS5syn-pTEF1-ΔF276A_HiTehA)
CEN.PK MA_tr_HiTehA-GFP	9169	*MATa pdc1, pdc5, pdc6, TEF1p-PYC1, PGK1p-PYC2, KlLEU2, TDH3p-MDH3Δskl, KlURA3, TEF1p-HiTehA-GFP, SpHIS5*	p0743 (pXI-1-loxP-KlLEU2-PYC1<-PTEF1-PPGK1->PYC2), p2860 (pX-2-loxP-KlURA-ScMdh3deltaSKL<-PTDH3), pCfB9027 (XII-4-HIS5syn-pTEF1-HiTehA-GFP)
CEN.PK MA_tr_ΔF276A_HiTehA-GFP	9172	*MATa pdc1, pdc5, pdc6, TEF1p-PYC1, PGK1p-PYC2, KlLEU2, TDH3p-MDH3Δskl, KlURA3, TEF1p--*ΔF276A_*HiTehA-GFP, SpHIS5*	p0743 (pXI-1-loxP-KlLEU2-PYC1<-PTEF1-PPGK1->PYC2), p2860 (pX-2-loxP-KlURA-ScMdh3deltaSKL<-PTDH3), pCfB9030 (XII-4-HIS5syn-pTEF1-ΔF276A_HiTehA-GFP)
CEN.PK MA_tr_AtALMT12	9160	*MATa pdc1, pdc5, pdc6, TEF1p-PYC1, PGK1p-PYC2, KlLEU2, TDH3p-MDH3Δskl, KlURA3, TEF1p-AtALMT12, SpHIS5*	p0743 (pXI-1-loxP-KlLEU2-PYC1<-PTEF1-PPGK1->PYC2), p2860 (pX-2-loxP-KlURA-ScMdh3deltaSKL<-PTDH3), pCfB9018 (XII-4-HIS5syn-pTEF1-*AtALMT12*)
CEN.PK MA_tr_AtALMT12-GFP	9167	*MATa pdc1, pdc5, pdc6, TEF1p-PYC1, PGK1p-PYC2, KlLEU2, TDH3p-MDH3Δskl, KlURA3, TEF1p-AtALMT12-GFP, SpHIS5*	p0743 (pXI-1-loxP-KlLEU2-PYC1<-PTEF1-PPGK1->PYC2), p2860 (pX-2-loxP-KlURA-ScMdh3deltaSKL<-PTDH3), pCfB9025 (XII-4-HIS5syn-pTEF1-*AtALMT12-GFP*)
CEN.PK MA_tr-ΔF329A_SpMAE1	8830-32	*MATa pdc1, pdc5, pdc6, TEF1p-PYC1, PGK1p-PYC2, KlLEU2, TDH3p-MDH3Δskl, KlURA3, TEF1p-SpMAE1, SpHIS5*	p0743 (pXI-1-loxP-KlLEU2-PYC1<-PTEF1-PPGK1->PYC2), p2860 (pX-2-loxP-KlURA-ScMdh3deltaSKL<-PTDH3), pCfB8126 (XII-4-HIS5syn-pTEF1-ΔF,329A-SpMae1)
CEN.PK MA_tr_ΔF107,329A_SpMAE1	8828	*MATa pdc1, pdc5, pdc6, TEF1p-PYC1, PGK1p-PYC2, KlLEU2, TDH3p-MDH3Δskl, KlURA3, TEF1p-SpMAE1, SpHIS5*	p0743 (pXI-1-loxP-KlLEU2-PYC1<-PTEF1-PPGK1->PYC2), p2860 (pX-2-loxP-KlURA-ScMdh3deltaSKL<-PTDH3), pCfB8689 (XII-4-HIS5syn-pTEF1-ΔF107,329A_SpMae1)
CEN.PK MA_tr_ΔCterminal_SpMAE1	8829	*MATa pdc1, pdc5, pdc6, TEF1p-PYC1, PGK1p-PYC2, KlLEU2, TDH3p-MDH3Δskl, KlURA3, TEF1p-*ΔCterminal_*SpMAE1, SpHIS5*	p0743 (pXI-1-loxP-KlLEU2-PYC1<-PTEF1-PPGK1->PYC2), p2860 (pX-2-loxP-KlURA-ScMdh3deltaSKL<-PTDH3), pCfB8690(XII-4-HIS5syn-pTEF1-ΔCterminal_SpMae1)
CEN.PK MA_tr-ΔF329A_SpMAE1-GFP	9174	*MATa pdc1, pdc5, pdc6, TEF1p-PYC1, PGK1p-PYC2, KlLEU2, TDH3p-MDH3Δskl, KlURA3, TEF1p-SpMAE1-GFP, SpHIS5*	p0743 (pXI-1-loxP-KlLEU2-PYC1<-PTEF1-PPGK1->PYC2), p2860 (pX-2-loxP-KlURA-ScMdh3deltaSKL<-PTDH3), pCfB9032 (XII-4-HIS5syn-pTEF1-ΔF329A-SpMae1-GFP)
CEN.PK MA_tr_ΔF107,329A_SpMAE1-GFP	9175	*MATa pdc1, pdc5, pdc6, TEF1p-PYC1, PGK1p-PYC2, KlLEU2, TDH3p-MDH3Δskl, KlURA3, TEF1p-SpMAE1-GFP, SpHIS5*	p0743 (pXI-1-loxP-KlLEU2-PYC1<-PTEF1-PPGK1->PYC2), p2860 (pX-2-loxP-KlURA-ScMdh3deltaSKL<-PTDH3), pCfB9033 (XII-4-HIS5syn-pTEF1-ΔF107,329A_SpMae1-GFP)
CEN.PK MA_tr_ΔCterminal_SpMAE1-GFP	9176	*MATa pdc1, pdc5, pdc6, TEF1p-PYC1, PGK1p-PYC2, KlLEU2, TDH3p-MDH3Δskl, KlURA3, TEF1p-*ΔCterminal_*SpMAE1-GFP, SpHIS5*	p0743 (pXI-1-loxP-KlLEU2-PYC1<-PTEF1-PPGK1->PYC2), p2860 (pX-2-loxP-KlURA-ScMdh3deltaSKL<-PTDH3), pCfB9034 (XII-4-HIS5syn-pTEF1-ΔCterminal_SpMae1-GFP)

Table S2. List of primers used in the study.

Primer name	Sequence	Application
Leader_fw	GGCTTAAUGTTTTTATTTTTAATTTTCTTTCAAATACTACCACCA	To amplify the ORAI1 leader sequence for cloning in the pUSER016
Leader_rv	GGTTTAAUGCTGAGGTTTAATTAAGCCTCAGCTTAAATGGCACCATAATTGTTGTAGAAGCAA	To amplify the ORAI1 leader sequence for cloning in the pUSER016
GFP_fw	GGCTTAAUATGTCTAAAGGTGAAGAATTATTCACTGGTGT	To amplify the GFP orf for cloning in the pUSER016
GFP_rv	GGTTTAAUTCATTTGTACAATTCATCCATACCATGGGTAATACCAGCAGCAGTA	To amplify the GFP orf for cloning in the pUSER016
GFP_fw.fusion	ATTACATTAUCCATGTCTAAAGGTGAAGAATTATTCACTGGTGT	To amplify the GFP orf for fusion fragments for cloning in the pUSER016
ScCTP1_fw	GGCTTAAUATGTCCAGTAAAGCTACCAAAAGTGACGTAGATCCA	To amplify the orf for cloning in the pUSER016
ScCTP1_rv	GGTTTAAUTCAGGCTAGCATAACTAAGACCTTTTCATAGATAGTGAA	To amplify the orf for cloning in the pUSER016
ScCTP1_fw.fusion	ATAATGTAAUGGCTAGCATAACTAAGACCTTTTCATAGATAGTGAA	To amplify the orf for fusion fragment for cloning in the pUSER016
EcDCUC_fw	GGCTTAAUATGCTGACATTCATTGAGCTCCTTATTGGGGTTGTGGTTATTGTGGGTGT	To amplify the orf for cloning in the pUSER016
EcDCUC_rv	GGTTTAAUTTACTTGCCTGTGACCGCTGCTGCCGTTCCTGGCACCATCAGCTCTGTAGCA	To amplify the orf for cloning in the pUSER016
EcDCUB_fw	GGCTTAAUATGTTATTTACTATCCAACTTATCATAATACTGATATGTCTGTTTTA	To amplify the orf for cloning in the pUSER016
EcDCUB_rv	GGTTTAAUTTATAAGAACCCGTACATCGCGGCGAAGATCCAGCCGAAGACGCACGAT	To amplify the orf for cloning in the pUSER016
EcEMRY_fw	GGCTTAAUATGGCAATCACTAAATCAACTCCGGCACCATTAACCGGTGGGACGTTA	To amplify the orf for cloning in the pUSER016
EcEMRY_rv	GGTTTAAUTCACCCAACGCCTTTCGCTGTAAACGGCGGTTTCGCAAACCAAACCAA	To amplify the orf for cloning in the pUSER016
EcYFC_fw	GGCTTAAUATGTCCGCAATCACTGAATCCAAACCAACAAGAAGATGGGCAATGCCCGAT	To amplify the orf for cloning in the pUSER016
EcYFC_rv	GGTTTAAUTTAGTGGTAGCCCATCAACTGAGCGCCGATCACTACGACGCTGGACAT	To amplify the orf for cloning in the pUSER016
ScMCH1_fw	GGCTTAAUATGCCTCTATCAAAGGTGGAGCACTACCTTTCATACCATACGCGCTTACTCT	To amplify the orf for cloning in the pUSER016
ScMCH1_rv	GGTTTAAUTTAAATTCTGAGTTTTCTACTTTTTAATTTCCAAAATACTACAGCTGAAAGA	To amplify the orf for cloning in the pUSER016
ScMCH2_fw	GGCTTAAUATGTCCGAAGAACGGCATGAAGATCATCATAGGGATGTTGAAAATAAATTGA	To amplify the orf for cloning in the pUSER016
ScMCH2_rv	GGTTTAAUTTAGACTCTCCTAGGTAATTGTTTATAACGAAAAAGACATTTACTCATATGT	To amplify the orf for cloning in the pUSER016

ScMCH3_fw	GGCTTAAUATGTCAACGCACTCAAACGACTACTTTTCTGCTTCTTCCGGAATGGTCTC	To amplify the orf for cloning in the pUSER016
ScMCH3_rv	GGTTTAAUCTAGACCTTCATTGGATATACCATTCTTAGGAAGTATTTTTAATGCCCGGA	To amplify the orf for cloning in the pUSER016
ScMCH4_fw	GGCTTAAUATGTTGAACATTCCCATAATTGCTAACTCCAAGAGGTTCCTGTTCTCAA	To amplify the orf for cloning in the pUSER016
ScMCH4_rv	GGTTTAAUTTAAAACTTACAAAGCTTCGCACCAACGCAAATATGCCTTGATATTATAT	To amplify the orf for cloning in the pUSER016
SpMAE1_fw	GGCTTAAUATGGGTGAACTCAAGGAAATCTTGAAACAGAGGTATCATGAGT	To amplify the orf for cloning in the pUSER016
SpMAE1_rv	GGTTTAAUTTAAACGCTTTCATGTTCACTACTAGGAGGATCCGATTCA	To amplify the orf for cloning in the pUSER016
AsDCT_fw	GGCTTAAUATGGATTTTTTGATGAATCTTAGTGAAGGTACACAGTTCACTATTCAACT	To amplify the orf for cloning in the pUSER016
AsDCT_rv	GGTTTAAUTTAAAGATAACCGTACAAGCCTGTAAAGATATAACCAAAGATACATGATGTA	To amplify the orf for cloning in the pUSER016
AsSLC13_fw	GGCTTAAUATGAATATCACTCCAGAACTTAAATCCAGATTTAATCCAAAGATCATCAT	To amplify the orf for cloning in the pUSER016
AsSLC13_rv	GGTTTAAUTTAATTCAACCAGAACAAGTAACCGAAAGTAGCGATGATAAAAATACTGA	To amplify the orf for cloning in the pUSER016
AoMAE1like_fw	GGCTTAAUATGAATATCGAGCTTCTTCATCCTAGACATATGCCAAAGCCCGGCAACGA	To amplify the orf for cloning in the pUSER016
AoMAE1like_rv	GGTTTAAUTTAAGCAGGACTATGATTGCCTGGAGATTCCGGCTTGATCTCATTGAT	To amplify the orf for cloning in the pUSER016
Cassette_fw	GTGCTGCAAGGCGATTAAGTTGGGTAACGC	For cassette amplification from Puser016
Cassette_rv	CCTCGAGGCGGCCGCCTGCAG	For cassette amplification from Puser016
PTEF1_fw	ACCTGCACUTTGTAATTAAAACTTAG	amplification of *TEF1(/PGK1)* promoter for cloning into integrative vector
PTEF1_rv	CACGCGAUGCACACACCATAGCTTC	amplification of *TEF1* promoter for cloning into integrative vector
PTEF1->_fw	CGTGCGAUGCACACACCATAGCTTC	amplification of *TEF1* promoter for cloning into integrative vector
PTEF1->_rv	ATGACAGAUTTGTAATTAAAACTTAG	amplification of *TEF1* promoter for cloning into integrative vector
PTDH3_fw	CACGCGAUATAAAAAACACGCTTTTTCAG	amplification of *TDH3* promoter for cloning into integrative vector
PTDH3_rv	ACCTGCACUTTTGTTTGTTTATGTGTGTTTATTC	amplification of *TDH3* promoter for cloning into integrative vector
PPGK1_rv	ATGACAGAUTTGTTTTATATTTGTTG	amplification of *PGK1/TEF1* promoter for cloning into integrative vector
ScPYC1_U1_fw	AGTGCAGGU AAAACA ATGTCGCAAAGAAAATTCG	amplification of *PYC1* gene for cloning into integrative vector

ScPYC1_U1_rv	CGTGCGAUTCATGCCTTAGTTTCAACAG	amplification of *PYC1* gene for cloning into integrative vector
ScPYC2_U2_fw	ATCTGTCAUAAAACAATGAGCAGTAGCAAGAAATTG	amplification of *PYC2* gene for cloning into integrative vector
ScPYC2_U2_rv	CACGCGAUTTACTTTTTTTGGGATGGG	amplification of *PYC2* gene for cloning into integrative vector
SpMae1_U2_fw	ATCTGTCAUAAAACAATGGGTGAACTCAAGGAAATC	amplification of *SpMae1* gene for cloning into integrative vector
SpMae1_U2_rv	CACGCGAUTTAAACGCTTTCATGTTCAC	amplification of *SpMae1* gene for cloning into integrative vector
ScMdh3deltaSKL_U1_fw	AGTGCAGGUAAAACAATGGTCAAAGTCGCAATTC	amplification of *MDH3* gene lacking peroxisomal targeting sequence gene for cloning into integrative vector
ScMdh3deltaSKL_U1_rv	CGTGCGAUTTAAGAGTCTAGGATGAAACTCTTG	amplification of *MDH3* gene lacking peroxisomal targeting sequence gene for cloning into integrative vector
RoMAElike_1fw	AGTGCAGGUAAAACAATGATTGAAAAAAAAAAGAAGGG	amplification of *R. oryzae* Mae like gene for cloning into integrative vector
RoMAElike_rev	CGTGCGAUCTACTGGACCATACTAAGGCAAG	amplification of *R. oryzae* Mae like gene for cloning into integrative vector
ScCTP1_2fw	ATCTGTCAUAAAACAATGTCCAGTAAAGCTACCAA	amplification of *CTP1* gene for cloning into integrative vector
ScCTP1_2rev	CACGCGAUTCAGGCTAGCATAACTAAGACCT	amplification of *CTP1* gene for cloning into integrative vector
ScCTP1leader_2fw	ATCTGTCAUAAAACAATGGAGGCTCTGTCTTGGAG	amplification of *CTP1* gene with membrane targeting sequence for cloning into integrative vector
EcDcuC_2fw	ATCTGTCAUAAAACAATGCTGACATTCATTGAGCT	amplification of *E. coli DcuC* gene for cloning into integrative vector
EcDcuC_2rev	CACGCGAUTTACTTGCCTGTGACCGCTGCTG	amplification of *E. coli DcuC* gene for cloning into integrative vector
EcDcuB_2fw	ATCTGTCAUAAAACAATGTTATTTACTATCCAACT	amplification of *E. coli DcuB* gene for cloning into integrative vector
EcDcuB_2rev	CACGCGAUTTATAAGAACCCGTACATCGCGG	amplification of *E. coli DcuB* gene for cloning into integrative vector
AsDcT_2fw	ATCTGTCAUAAAACAATGGATTTTTTGATGAATCT	amplification of *A. succinogenes DcT* gene for cloning into integrative vector

AsDcT_2rev	CACGCGAUTTAAAGATAACCGTACAAGCCTG	amplification of *A. succinogenes DcT* gene for cloning into integrative vector
AsSlc13_2fw	ATCTGTCAUAAAACAATGAATATCACTCCAGAACT	amplification of *A. succinogenes Slc13* gene for cloning into integrative vector
AsSlc13_2rev	CACGCGAUTTAATTCAACCAGAACAAGTAAC	amplification of *A. succinogenes Slc13* gene for cloning into integrative vector
AoMAE1like_2fw	ATCTGTCAUAAAACAATGAATATCGAGCTTCTTCA	amplification of *A. oryzae Mae-like* gene for cloning into integrative vector
AoMAE1like_2rev	CACGCGAUTTAAGCAGGACTATGATTGCCTG	amplification of *A. oryzae Mae-like* gene for cloning into integrative vector
PR-24452 (AcDCT_fwd)	ATCTGTCAUAAAACAATGCATGTTCATGATACATTACCAGGTTCT TC	amplification of *A. carbonarius DcT* gene for cloning into integrative vector
PR-24540 (Seq_AcDCTGFP)	TCTGGTACTATCGCTTCTGT	amplification of *A. carbonarius DcT* gene for cloning into integrative vector
ADH1_test_fw	GAAATTCGCTTATTTAGCCGTGTC	amplification of genes for cloning into integrative vector
PR-24456 (AtSLAC1_fwd)	ATCTGTCAUAAAACAATGGAAAGAAAGCAATCTAATGCTCATTC TACTTTT	amplification of *A. thaliana SLAC1* gene for cloning into integrative vector
PR-24543 (Seq_AtSLAC1GFP_1)	GCGACCAATTTCTTGCATATTA	amplification of *A. thaliana SLAC1* gene for cloning into integrative vector
PR-24544 (Seq_AtSLAC1GFP_2)	GATGGTTGTTCTAGAACTTGTTT	amplification of *A. thaliana SLAC1* gene for cloning into integrative vector
PR-24464 (link_GFP_fwd)	AGCTTCTGGUGCAATGTCTAAAGGTGAAGAATTATTCACTGGTGT TG	amplification of *GFP fusion* genes for cloning into integrative vector
PR-24458 (HiTehA_fwd)	ATCTGTCAUAAAACAATGTTGCATTTCGCTCATATATTTCAAAAT AAAGTGC	amplification of *H. influenzae TehA* genes for cloning into integrative vector
PR-24545 (Seq_HiTehAGFP)	TTATCTATTCTTCGGTGCCG	amplification of *H. influenzae TehA* genes for cloning into integrative vector
PR-24464 (link_GFP_fwd)	AGCTTCTGGUGCAATGTCTAAAGGTGAAGAATTATTCACTGGTGT TG	amplification of *H. influenzae TehA* genes for cloning into integrative vector
PR-24454 (AtALMT12_fwd)	ATCTGTCAUAAAACAATGTCAAATAAAGTTCATGTTGGTTCATTA GAAATGGA	amplification of *A. thaliana ALMT12 TehA* genes for cloning into integrative vector
PR-24542 (Seq_AtALMT12GFP_2)	TTGCAAAATACTGAAACTGGTAC	amplification of *A. thaliana ALMT12 TehA* genes for cloning into integrative vector

PR-24541 (Seq_AtALMT12GFP_1	AGATTTGCATAAAACCACTGTC	amplification of *A. thaliana ALMT12 TehA* genes for cloning into integrative vector
PR-24472 (SpMAE_fwd)	ATCTGTCAUAAAACAATGGGTGAACTCAAGG	amplification of *SpMae1* gene for cloning into integrative vector
PR-24539 (Seq_SpMAEGFP)	GGTTTATCTTTTACTGTTTGCC	amplification of *SpMae1* gene for cloning into integrative vector
ADH1_test_fw	GAAATTCGCTTATTTAGAAGTGTC	verification of gene expression cassette cloning into EasyClone vectors
CYC1_test_rv	CTCCTTCCTTTTCGGTTAGAG	verification of gene expression cassette cloning into EasyClone vectors
ID2220_vec_DW_out	CCTGCAGGACTAGTGCTGAG	verification of EasyClone vector chromosomal integration
ID2221_vec_UP_out	GTTGACACTTCTAAATAAGCGAATTTC	verification of EasyClone vector chromosomal integration
ID901 X-2-up-out-sq	TGCGACAGAAGAAAGGGAAG	verification of site X-2 chromosomal integration
ID902 X-2-down-out-sq	GAGAACGAGAGGACCCAACAT	verification of site X-2 chromosomal integration
ID907 XI-1-up-out-sq	CTTAATGGGTAGTGCTTGACACG	verification of site XI-1 chromosomal integration
ID908 XI-1-down-out-sq	GAAGACCCATGGTTCCAAGGA	verification of site XI-1 chromosomal integration
ID897 XII-4-up-out-sq	GAACTGACGTCGAAGGCTCT	verification of site X-4 chromosomal integration
ID898 XII-4-down-out-sq	CGTGAAATCTCTTTGCGGTAG	verification of site X-4 chromosomal integration

Table S3. List of plasmids used in the study.

Plasmid name	Integration site/replicon	Yeast selection marker	Insert	Parental vector	Cloned BioBricks	Source
pUSER016-Mae1	ori	-	T7p→SpMAE1	-	-	This study
pUSER016-Ctp1	ori	-	T7p→ScCTP1	-	-	This study
pUSER016-L5.Ctp1	ori	-	T7p→Leader_CTP1	-	-	This study
pUSER016-DcuC	ori	-	T7p→EcDCUC	-	-	This study
pUSER016-DcuB	ori	-	T7p→EcDCUB	-	-	This study
pUSER016-DcT	ori	-	T7p→AsDCT	-	-	This study

pUSER016-Slc13	ori	-	T7p→AsSLC13	-	-	This study
pUSER016-Mae1like	ori	-	T7p→AoMAE1like	-	-	This study
pUSER016-Mch1	ori	-	T7p→ScMCH1	-	-	This study
pUSER016-Mch2	ori	-	T7p→ScMCH2	-	-	This study
pUSER016-Mch3	ori	-	T7p→ScMCH3	-	-	This study
pUSER016-Mch4	ori	-	T7p→ScMCH4	-	-	This study
pUSER016-YfcC	ori	-	T7p→EcYFCC	-	-	This study
pUSER016-Emry	ori	-	T7p→EcEMRY	-	-	This study
pCfB388	XI-1	LEU	-	-	-	(16)
pCfB255	X-2	URA	-	-	-	(16)
pCfB2228	XII-4	HIS	-	-	-	(17)
p0743	XI-1	LEU	PYC1←TEF1p-PGK1p→PYC2	pCfB388	BB0149 (ScPYC1<-), BB0150 (ScPYC2->), BB0010 (<-PTEF1-PPGK1->)	This study
p2860	X-2	URA	MDH3Δskl←TDH3p	pCfB255	BB0794 (ScMdh3deltaSKL<-PTDH3)	This study
pCfB6547	XII-4	HIS	TEF1p→SpMAE1	pCfB2228	BB0301 (PTEF1->), BB2071 (SpMae1_2)	This study
pCfB8126	XII-4	HIS	TEF1p→ΔF329A-SpMae1	pCfB2228	BB0301 (PTEF1->), BB2071 (SpMae1_2)	This study
pCfB8689	XII-4	HIS	TEF1p→ΔF107,329A_SpMae1	pCfB2228		This study
pCfB8690	XII-4	HIS	TEF1p-ΔCterminal_SpMae1	pCfB2228		This study
pCfB6548	XII-4	HIS	RoMAElike←TEF1p	pCfB2228		This study
pCfB6549	XII-4	HIS	TEF1p→CTP1	pCfB2228	BB0301 (PTEF1->), BB2073 (ScCTP1)	This study
pCfB6550	XII-4	HIS	TEF1p→CTP1leader	pCfB2228	BB0301 (PTEF1->), BB2074 (ScCTP1leader)	This study
pCfB6551	XII-4	HIS	TEF1p→EcDCUC	pCfB2228	BB0301 (PTEF1->), BB2075 (EcDcuC)	This study
pCfB6552	XII-4	HIS	TEF1p→EcDCUB	pCfB2228	BB0301 (PTEF1->), BB2076 (EcDcuB)	This study
pCfB6553	XII-4	HIS	TEF1p→AsDCT	pCfB2228	BB0301 (PTEF1->), BB2077 (AsDcT)	This study
pCfB6554	XII-4	HIS	TEF1p→AsSLC13	pCfB2228	BB0301 (PTEF1->), BB2078 (AsSlc13)	This study
pCfB6555	XII-4	HIS	TEF1p→AoMAE1like	pCfB2228	BB0301 (PTEF1->), BB2079 (AoMAE1like)	This study
p1977	ori	-	TDH3p	-	-	(17)
pSP-GM1	ori, 2μ	KlURA3	TEF1p	-	-	(18)
pCfB9017	XII-4	HIS	TEF1p→ AcDCT	pCfB2228	BB3970, BB3970, BB3971(AcDCT)	This study
pCfB9021	XII-4	HIS	TEF1p→ ΔF354A_AcDCT	pCfB2228	BB3970, BB3980 (AcDCT_mut_part1), BB3981 (AcDCT_mut_part2)	This study
pCfB9024	XII-4	HIS	TEF1p→ AcDCT-GFP	pCfB2228	BB3970, BB3975 (AcDCT_GFPlinker), BB3979 (Linker_GFP_gene2)	This study

pCfB9028	XII-4	HIS	TEF1p→ ΔF354A_AcDCT-GFP	pCfB2228	BB3970, BB3980(AcDCT_mut_part1), BB3986(AcDCT_mut_GFPlinker),BB0397(ACH1-vo3_UP)	This study
pCfB9019	XII-4	HIS	TEF1p→ AtSLAC1	pCfB2228	BB3970, BB3973 (AtSLAC1_gene2)	This study
pCfB9022	XII-4	HIS	TEF1p→ ΔF450A_AtSLAC1	pCfB2228	BB3970, BB3982 (AtSLAC1_mut_part1), BB3983 (AtSLAC1_mut_part2)	This study
pCfB9026	XII-4	HIS	TEF1p→ AtSLAC1-GFP	pCfB2228	BB3970, BB3977 (AtSLAC1_GFPlinker), BB3979 (Linker_GFP_gene2)	This study
pCfB9029	XII-4	HIS	TEF1p→ ΔF450A_AtSLAC1-GFP	pCfB2228	BB3970, BB3982 (AtSLAC1_mut_part1), BB3987 (AtSLAC1_mut_GFPlinker), BB0397(ACH1-evo3_UP)	This study
pCfB9020	XII-4	HIS	TEF1p→ HiTehA	pCfB2228	BB3970, BB3974 (HiTehA_gene2)	This study
pCfB9023	XII-4	HIS	TEF1p→ ΔF276A_ HiTehA	pCfB2228	BB3970, BB3984 (HiTehA_mut_part1), BB3985 (HiTehA_mut_part2)	This study
pCfB9027	XII-4	HIS	TEF1p→ HiTehA -GFP	pCfB2228	BB3970, BB3978 (HiTehA_GFPlinker), BB3979 (Linker_GFP_gene2)	This study
pCfB9030	XII-4	HIS	TEF1p→ ΔF276A_ HiTehA -GFP	pCfB2228	BB3970, BB3984 (HiTehA_mut_part1), BB3988 (HiTehA_mut_GFPlinker), BB0397(ACH1-evo3_UP)	This study
pCfB9018	XII-4	HIS	TEF1p→*AtALMT12*	pCfB2228	BB3970, BB3972 (AtALMT12_gene2)	This study
pCfB9025	XII-4	HIS	TEF1p→ *AtALMT12-GFP*	pCfB2228	BB3970, BB3976 (AtALMT12_GFPlinker), BB3979 (Linker_GFP_gene2)	This study
pCfB9032	XII-4	HIS	TEF1p→ ΔF329A-SpMae1-GFP	pCfB2228	BB3970, BB3990 (SpMAE_F329A_GFPlinker), BB3979 (Linker_GFP_gene2)	This study
pCfB9033	XII-4	HIS	TEF1p→ ΔF107,329A-SpMae1-GFP	pCfB2228	BB3970, BB3991 (SpMAE_F329A_F109A_GFPlinker), BB3979 (Linker_GFP_gene2)	This study
pCfB9034	XII-4	HIS	TEF1p→ ΔCterminal_SpMae1-GFP	pCfB2228	BB3970, BB3992 (SpMAE_Cterm_GFPlinker), BB3979 (Linker_GFP_gene2)	This study

Table S4. List of DNA BioBricks.

Name	Descriptive name	Primer pair for PCR	Template for PCR
BB0008	PTEF1←	PTEF1_fw, PTEF1_rv	pSP-GM1

BB0301	PTEF1→	PTEF1->_fw, PTEF1->_rv	pSP-GM1
BB0410	PTDH3←	PTDH3_fw, PTDH3_rv	p1977 (pUC19-PTDH3-PTEF1)
BB0010	←PTEF1-PPGK1→	PTEF1_fw, PPGK1_rv	pSP-GM1
BB0149	ScPYC1←	ScPYC1_U1_fw, ScPYC1_U1_rv	*S. cerevisiae* genomic DNA
BB0150	ScPYC2→	ScPYC2_U2_fw, ScPYC2_U2_rv	*S. cerevisiae* genomic DNA
BB0546	SpMae1→	SpMAE1_U2_fw, SpMAE1_U2_rv	*Sch. pombe* genomic DNA
BB0537	ScMDH3ΔSKL←	ScMDH3ΔSKL_U1_fw, ScMDH3ΔSKL_U1_rv	*S. cerevisiae* genomic DNA
BB2073	ScCTP1	ScCTP1_2fw, ScCTP1_2rev	pUSER016-Ctp1 no.3
BB2074	ScCTP1leader	ScCTP1leader_2fw, ScCTP1_2rev	pUSER016-L5.Ctp1 no.4
BB2075	EcDCUC	EcDCUC_2fw, EcDCUC_2rev	p016-DcuC no. 4-4
BB2076	EcDCUB	EcDCUB_2fw, EcDCUB_2rev	p016-DcuB no. 3
BB2077	AsDCT	AsDCT_2fw, AsDCT_2rev	p016-DcT no. 1
BB2078	AsSLC13	AsSLC13_2fw, AsSLC13_2rev	p016-Slc13 no. 4
BB2079	AoMAE1like	AoMAE1like_2fw, AoMAE1like_2rev	p016-Mae1like no. 2
BB3970	TEFp-DicarboxTrans	PR-24450 (TEF1p_fwd), PR-24451 (TEF1p_rev)	pCfB6547 (XII-4-HIS5syn-pTEF1-SpMae1)
BB3979	Linker_GFP_gene2	PR-24464 (link_GFP_fwd) PR-24465 (yeGFP_rev)	pCfB8801 (X-2-MarkerFree-GFPcterm-MsErgT<-TEF1)
BB3992	SpMAE_Cterm_GFPlinker	PR-24472 (SpMAE_fwd) PR-24473 (SpMAE_GFPlink_rev)	pCFB8690(p6547 -c terminal)
BB3991	SpMAE_F329A_F109A_GFPlinker	PR-24472 (SpMAE_fwd) PR-24473 (SpMAE_GFPlink_rev)	pCFB8689(p8126_F107A no.1)
BB3990	SpMAE_F329A_GFPlinker	PR-24472 (SpMAE_fwd) PR-24473 (SpMAE_GFPlink_rev)	pcfB8126(p6547-SpMAE1329F/A)
BB3976	AtALMT12_GFPlinker	PR-24454 (AtALMT12_fwd) PR-24461 (AtALMT12_GFPlink_rev)	pCfB8911 (AtALMT12)
BB3972	AtALMT12_gene2	PR-24454 (AtALMT12_fwd) PR-24455 (AtALMT12_rev)	pCfB8911 (AtALMT12)
BB3984	HiTehA_mut_part1	PR-24458 (HiTehA_fwd) PR-24470 (HiTehA_mut_rev)	pCfB8913 (HiTehA)
BB3988	HiTehA_mut_GFPlinker	PR-24471 (HiTehA_mut_fwd) PR-24463 (HiTehA_GFPlink_rev)	pCfB8913 (HiTehA)
BB0397	ACH1-evo3_UP		
BB3978	HiTehA_GFPlinker	PR-24458 (HiTehA_fwd) PR-24463 (HiTehA_GFPlink_rev)	pCfB8913 (HiTehA)
BB3985	HiTehA_mut_part2	PR-24471 (HiTehA_mut_fwd) PR-24459 (HiTehA_rev)	pCfB8913 (HiTehA)
BB3984	HiTehA_mut_part1	PR-24458 (HiTehA_fwd) PR-24470 (HiTehA_mut_rev)	pCfB8913 (HiTehA)
BB3974	HiTehA_gene2	PR-24458 (HiTehA_fwd) PR-24459 (HiTehA_rev)	pCfB8913 (HiTehA)
BB3987	AtSLAC1_mut_GFPlinker	PR-24469 (AtSLAC1_mut_fwd) PR-24462 (AtSLAC1_GFPlink_rev)	pCfB8912 (AtSLAC1)
BB3982	AtSLAC1_mut_GFPlinker	PR-24469 (AtSLAC1_mut_fwd) PR-24462 (AtSLAC1_GFPlink_rev)	pCfB8912 (AtSLAC1)
BB3977	AtSLAC1_GFPlinker	PR-24456 (AtSLAC1_fwd) PR-24462 (AtSLAC1_GFPlink_rev)	pCfB8912 (AtSLAC1)
BB3983	AtSLAC1_mut_part2	PR-24469 (AtSLAC1_mut_fwd) PR-24457 (AtSLAC1_rev)	pCfB8912 (AtSLAC1)
BB3980	AcDCT_mut_part1	PR-24452 (AcDCT_fwd) PR-24466 (AcDCT_mut_rev)	pCfB8910 (AcDCT)
BB3986	AcDCT_mut_GFPlinker	PR-24467 (AcDCT_mut_fwd) PR-24460 (AcDCT_GFPlink_rev)	pCfB8910 (AcDCT)
BB3973	AtSLAC1_gene2	PR-24456 (AtSLAC1_fwd) PR-24457 (AtSLAC1_rev)	pCfB8912 (AtSLAC1)

BB3981	AcDCT_mut_part2	PR-24467 (AcDCT_mut_fwd)<br>PR-24453 (AcDCT_rev)	pCfB8910 (AcDCT)
BB3975	AcDCT_GFPlinker	PR-24452 (AcDCT_fwd)<br>PR-24460 (AcDCT_GFPlink_rev)	pCfB8910 (AcDCT)
BB3971	AcDCT_Gene2	PR-24452 (AcDCT_fwd)<br>PR-24453 (AcDCT_rev)	pCfB8910 (AcDCT)

Table S5. Transitions for the TCA intermediates analyzed.

Compound	Rt, min	Transition	Collision energy
Malic acid	0.92	1. 133→116<br>2. 133→71	12<br>16
Succinic acid	1.67	1. 117→80<br>2. 117→99	12<br>12
Fumaric acid	1.66	1. 115→71<br>2. 115→32	8<br>20
Citric acid	1.67	1. 191→111<br>2. 191→86	13<br>17
Glyoxylic acid	0.60	1. 73→45<br>(2. 73→73)	15
Alfaketoglutaric acid	1.14	1. 145→101<br>2. 145→57	9<br>11
Isocitric acid	0.96	1. 191→111<br>2. 191→73	12<br>17

Table S6. Accession numbers of the sequences.

Species	Accession
Quercus suber	XP_023892255 (QsMCH1), POE47723 (QsMCH3), XP_023902613 (QsMCH2), XP_023877431 (QsMCH4), XP_023900349 (QsMAE1), and POE94461 (QsJEN1)
Trichuris trichiura	CDW56989 (TtDCT), CDW56989 (TtDCUB), and CDW58066 (TtADY2)
Nephila clavipes	PRD28662 (NcSLC13), and PRD18006 (NcYFCC)
Beauveria bassiana D1-5	KGQ13609 (BbEMRY), and KGQ11260 (BbDCUC)
Marmota marmota marmota	MCH2 (MmMCH2): XP_015331577, and XP_015331484 (MmJEN1)
Crassostrea gigas	MCH1: XP_011422711
Deltaproteobacteria RIFCSPLOWO2_12_FULL_60_19	MCH2 (DrMCH2): OGQ80668
Branchiostoma belcheri	MCH3 (BbMCH3): XP_019617991
Bacterium SCGC AG-212-C10	MCH3 (BsMCH3): OAI40955
Seriola lalandi dorsalis	MCH4 (SlMCH4): XP_023276592
Chlamydia trachomatis	MCH4 (CtMCH4): CQB89607
Hordeum vulgare	CTP1 (HvCTP1): BAJ99694
Salvelinus alpinus	CTP1 (SaCTP1): XP_023867225
Streptomyces HGB0020	JEN1 (ShJEN1): WP_016433772
Chlamydia trachomatis	ADY2 (CtADY2): CQB88822
Marchantia polymorpha	ADY2 (MpADY2): OAE24668
Lucilia cuprina	DCUC (LcDCUC): XP_023307619
Enterocytozoon bieneusi	H348 SLC13 (EbSLC13): EED43380
Capsella rubella	SLC13 (CrSLC13): XP_006281644
Cajanus cajan	EMRY (CcEMRY): KYP77246
Plutella xylostella	EMRY (PxEMRY): XP_011567473
Arabidopsis thaliana	AT1G12480 (At_SLAC1), AT1G62280 (AtSLAH1), AT4G27970 (AtSLAH2), AT5G24030 (AtSLAH3), AT1G62262 (AtSLAH4), AT4G17970 (AtALMT12), AT2G17470 (AtALMT6), and AT3G18440 (AtALMT9)
Actinobacillus succinogenes	Asuc_1568/WP_012073299 (AsSLC13) and Asuc_1999/ WP_012073722 (AsDCT)

Escherichia coli	EG11883 (EcTEHA), EcK0614 (EcDCUC) (EcoGene accession: EG13545), EcK4116 (EcDCUB) (EcoGene accession: EG10006), EMRY (EcoGene accession: EG13283), and YFCC (EcoGene accession: EG12607)
Saccharomyces cerevisiae	YBR291C (ScCTP1, Genbank accession: NP_009850), YPL092W (ScSSU1), YKL217W (ScJEN1), YDL054C (ScMCH1), YKL221W (ScMCH2), and YCR010C (ScADY2)
Schizosaccharomyces pombe	NP_594777 (SpMAE1), NP_587754 (SpMAE1like),
Aspergillus fumigatus	MAE1 (AfMAE1): XP_747269.1
Aspergillus oryzae	MAE1 (AoMAE1like): Aor_1_62044/AO090206000038
Brassica rapa	SLAC1 (BrSLAC1): XP_009148588
Haemophilus influenzae	TEHA (HiTEHA): WP_010868994
Nicotiana attenuata	SLAC1 (NtSLAC1): XP_019252311
Schizosaccharomyces japonicus	MAE1 (S.japonicus MAE1): XP_002172654
Schizosaccharomyces octoporus	MAE1 (S.octoporus MAE1): XP_013018508
Schizosaccharomyces cryophilus	MAE1 (S.cryophilus MAE1): XP_013022427
Aspergillus carbonarius	DcT (MAE1): AQW79505.1

References

1. Verduyn C, Postma E, Scheffers WA, Van Dijken JP (1992) Effect of benzoic acid on metabolic fluxes in yeasts: a continuous-culture study on the regulation of respiration and alcoholic fermentation. *Yeast Chichester Engl* 8(7):501–517.
2. Zelle RM, Hulster E de, Kloezen W, Pronk JT, Maris AJA van (2010) Key Process Conditions for Production of C4 Dicarboxylic Acids in Bioreactor Batch Cultures of an Engineered Saccharomyces cerevisiae Strain. *Appl Environ Microbiol* 76(3):744–750.
3. van Maris AJA, et al. (2004) Directed Evolution of Pyruvate Decarboxylase-Negative Saccharomyces cerevisiae, Yielding a C2-Independent, Glucose-Tolerant, and Pyruvate-Hyperproducing Yeast. *Appl Environ Microbiol* 70(1):159–166.
4. Gietz RD, Woods RA (2006) Yeast transformation by the LiAc/SS Carrier DNA/PEG method. *Methods Mol Biol Clifton NJ* 313:107–120.
5. Nour-Eldin HH, Geu-Flores F, Halkier BA (2010) USER cloning and USER fusion: the ideal cloning techniques for small and big laboratories. *Methods Mol Biol Clifton NJ* 643:185–200.
6. Darbani B, et al. (2016) The biosynthetic gene cluster for the cyanogenic glucoside dhurrin in *Sorghum bicolor* contains its co-expressed vacuolar MATE transporter. *Sci Rep* 6:37079.
7. Clémençon B, Fine M, Schneider P, Hediger MA (2015) Rapid method to express and purify human membrane protein using the Xenopus oocyte system for functional and low-resolution structural analysis. *Methods Enzymol* 556:241–265.
8. Jones AM, et al. (2014) Border Control—A Membrane-Linked Interactome of Arabidopsis. *Science* 344(6185):711–716.
9. Finn RD, Clements J, Eddy SR (2011) HMMER web server: interactive sequence similarity searching. *Nucleic Acids Res* 39(Web Server issue):W29-37.
10. Krogh A, Larsson B, von Heijne G, Sonnhammer EL (2001) Predicting transmembrane protein topology with a hidden Markov model: application to complete genomes. *J Mol Biol* 305(3):567–580.
11. Spyropoulos IC, Liakopoulos TD, Bagos PG, Hamodrakas SJ (2004) TMRPres2D: high quality visual representation of transmembrane protein models. *Bioinforma Oxf Engl* 20(17):3258–3260.
12. Kelley LA, Mezulis S, Yates CM, Wass MN, Sternberg MJE (2015) The Phyre2 web portal for protein modeling, prediction and analysis. *Nat Protoc* 10(6):845–858.

13. Chen Y-H, et al. (2010) Homologue structure of the SLAC1 anion channel for closing stomata in leaves. *Nature* 467(7319):1074–1080.
14. Xu J, Zhang Y (2010) How significant is a protein structure similarity with TM-score = 0.5? *Bioinforma Oxf Engl* 26(7):889–895.
15. Whelan S, Goldman N (2001) A general empirical model of protein evolution derived from multiple protein families using a maximum-likelihood approach. *Mol Biol Evol* 18(5):691–699.
